# Taxonomic Chauvinism Revisited: Insight from Parental Care Research

**DOI:** 10.1371/journal.pone.0024192

**Published:** 2011-08-31

**Authors:** Zachary R. Stahlschmidt

**Affiliations:** Arizona State University, Tempe, Arizona, United States of America; University of Bristol, United Kingdom

## Abstract

Parental care (any non-genetic contribution by a parent that appears likely to increase the fitness of its offspring) is a widespread trait exhibited by a broad range of animal taxa. In addition to influencing the fitness of parent(s) and offspring, parental care may be inextricably involved in other evolutionary processes, such as sexual selection and the evolution of endothermy. Yet, recent work has demonstrated that bias related to taxonomy is prevalent across many biological disciplines, and research in parental care may be similarly burdened. Thus, I used parental care articles published in six leading journals of fundamental behavioral sciences (*Animal Behaviour*, *Behavioral Ecology*, *Behavioral Ecology and Sociobiology*, *Ethology*, *Hormones and Behavior*, and *Physiology & Behavior*) from 2001–2010 (*n* = 712) to examine the year-to-year dynamics of two types of bias related to taxonomy across animals: (1) taxonomic bias, which exists when research output is not proportional to the frequency of organisms in nature, and (2) taxonomic citation bias, which is a proxy for the breadth of a given article—specifically, the proportion of articles cited that refer solely to the studied taxon. I demonstrate that research on birds likely represents a disproportionate amount of parental care research and, thus, exhibits taxonomic bias. Parental care research on birds and mammals also refers to a relatively narrow range of taxonomic groups when discussing its context and, thus, exhibits taxonomic citation bias. Further, the levels of taxonomic bias and taxonomic citation bias have not declined over the past decade despite cautionary messages about similar bias in related disciplines— in fact, taxonomic bias may have increased. As in Bonnet et al. (2002), my results should not be interpreted as evidence of an ‘ornithological Mafia’ conspiring to suppress other taxonomic groups. Rather, I generate several rational hypotheses to determine why bias persists and to guide future work.

## Introduction

Parental care (any non-genetic contribution by a parent that appears likely to increase the fitness of its offspring) is of paramount importance, as it is a convergent trait used by a broad range of taxa [1]. Despite its benefits to offspring, parental care often reduces aspects of parental fitness (e.g., future reproductive efforts and longevity), which allows natural selection to mediate this parent-offspring tradeoff. Also, parental care is often inextricably involved in other evolutionary processes. For instance, the degree of parental investment often dictates the degree and direction of sexual selection (e.g., large, brightly colored male birds and female fish compete for mates with high parental investment) [1], [2]. Further, parental control of the developmental environment may play a role in the evolution of endothermy [3].

Behavioral biologists have a long history of investigating the ultimate and proximate mechanisms of parental care (reviewed in [1]). To best understand the dynamics of parental care, research should operate on two tenets: (1) researchers should explore parental care across animal taxa, and (2) researchers should devote relatively more attention to taxa that are particularly suitable parental care models. However, although a goal of all scientific research is objectivity, evidence in the past decade demonstrates that biological research generally involves some level of bias related to taxonomy [4]–[7]. The cause of such bias may be due to the methodological handicaps associated with working on certain taxa [6], taxonomic chauvinism [4], or both.

Two common types of bias related to taxonomy include taxonomic bias and taxonomic citation bias. Taxonomic bias exists when research output is not proportional to the frequency of organisms in nature, and it has been exhibited by biological disciplines ranging from ecology and evolution [4], [6] to animal conservation [5], [7]. In addition, taxonomic citation bias is a proxy for the breadth of a given article, specifically the proportion of articles cited that refer solely to the studied taxon. Significant taxonomic citation bias is exhibited in ecology and evolution [4] and animal behavior, in general [8].

Given its relatively recent attention, the degree of bias related to taxonomy and potential explanations for its persistence in parental care research remain unclear. Thus, I addressed several questions to fill this knowledge gap related to taxonomic groups, specifically mammals, birds, reptiles, amphibians, fish, and invertebrates. First, does taxonomic bias exist in parental care research, and have patterns of bias changed over the past decade? One would expect increasing parity in taxonomic representation in parental care research if researchers heeded cautionary messages about taxonomic bias in related disciplines (e.g., [4], [5], [7], [8]). Second, to what degree does taxonomic citation bias exist in parental care research, and does the specific journal or taxonomic group explain variation in this bias? Bias in parental care research may be less than, equivalent to, or greater than bias in other areas of animal behavior. Last, why does bias related to taxonomy persist in parental care research? I summarize several possible hypotheses that may explain why bias continues to exist.

## Methods

I used parental care articles published in by six leading journals of fundamental behavioral sciences (*Animal Behaviour*, *Behavioral Ecology*, *Behavioral Ecology and Sociobiology*, *Ethology*, *Hormones and Behavior*, and *Physiology & Behavior*) from 2001–2010 for all analyses. Initially, I used ISI Web of Knowledge to search for articles dealing with parental care from 2001–2010 in March 2011 (Topic = parent*). After I eliminated articles that did not entail parental care, the final sample size was 712 articles, including those involving brood parasitism, parent-offspring communication, and cooperative or communal breeding if the parent(s) provided care (see Supporting Information S1). Data met the appropriate assumption of parametric statistics, were transformed as necessary, or were analyzed using a non-parametric test (e.g., Spearman rank correlation test). I analyzed data using SPSS Statistics (version 15.0, SPSS, Inc., Chicago, IL, USA). I determined experiment-wise, two-tailed significance at α<0.05 for all tests and report all values as mean±s.e.m.

I used the entire sample of articles to examine taxon-based differences in the annual number of parental care articles published during the 10-year sampling period. Specifically, I used analyses of covariance (ANCOVA) with taxon and journal as main effects, taxon*journal as an interaction, and the annual number of articles published as a covariate. To examine taxonomic citation bias within each taxonomic group, I compared bias among randomly sub-sampled articles from each taxonomic group (*n* = 20) from the decade-long sample of parental care articles described above. Specifically, I used analyses of variance (ANOVA) with taxon and journal as main effects, year as a random effect, and taxon*journal as an interaction. Due to low sample size, I pooled articles on amphibian and reptilian parental care for analyses of taxonomic citation bias. Yet, the final sample size for this group was still lower than other groups (*n* = 15). For all ANCOVA and ANOVA tests, I initially ran the complete model (i.e., all effects, interactions, and covariates, if applicable) and parsimoniously removed variables of *α*>0.10. I then re-analyzed the data using the remaining variables to create the most robust model possible, which I report.

I used simple linear regression analyses or Spearman rank correlation tests to determine relationships between variables of interest (e.g., the year of sampling and the number of parental care articles published for each taxonomic group). To determine the degree of taxonomic citation bias in parental care research in relation to other areas of animal behavior, I pooled bias data from the sub-sample described above (*n* = 95) and used two-sample *t* tests to compare it to data from [8], which examined taxonomic citation bias in a sub-sample of 38 articles published in *Ethology*.

## Results

**Figure 1:**
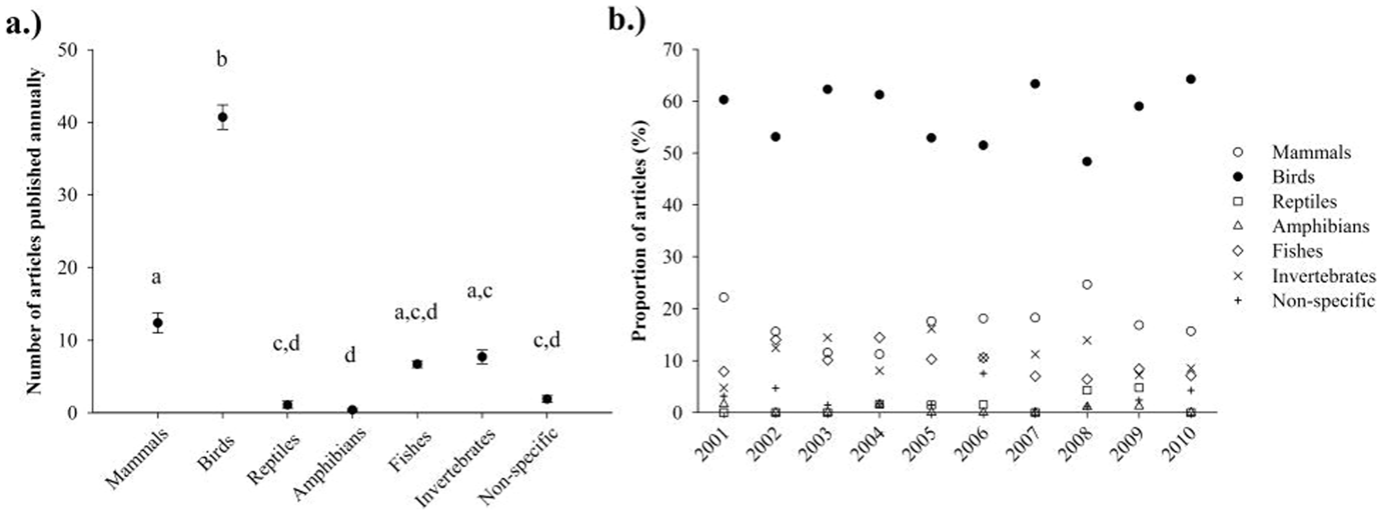
Taxonomic representation of parental care research in *Animal Behaviour*, *Behavioral Ecology*, *Behavioral Ecology and Sociobiology*, *Ethology*, *Hormones and Behavior*, and *Physiology & Behavior* 2001–2010 (*n* = 712). a.) The mean number of articles per year for five animal taxonomic groups, as well as parental care articles that were not taxon-specific. Values are displayed as mean±s.e.m., and significant differences among groups are denoted by letters (a≠b≠c≠d). b.) Year-to-year composition of parental care research based on major animal taxonomic group. No significant trends with time existed for any taxonomic group.

The composition of parental care articles was significantly influenced by taxon (*F*_6,356_ = 227, *P*<0.001), the interaction between journal and taxon (*F*_30,356_ = 27.0, *P*<0.001), and the annual number of articles published (covariate: *F*_1,356_ = 51.6, *P*<0.001). Specifically, parental care research was dominated by studies focusing on birds over the past decade (Fig. 1a). In fact, 412 (58%) of the articles focused on avian parental care, and birds were the most popular parental care model every year (Fig. 1b). A significant positive relationship between year and the total number of parental care papers existed (*F*_1,8_ = 5.5, *R* ^2^ = 0.41, *P* = 0.046). Further, a significant positive relationship between year and the number of bird-specific papers existed (*F*_1,8_ = 6.8, *R* ^2^ = 0.46, *P* = 0.031). However, the proportion of papers on birds was not significantly related to year (Fig. 1b; *F*_1,8_ = 0.013, *R* ^2^ = 0.46, *P* = 0.91) (note: this analysis violates an assumption of parametric statistics, namely independence). No significant relationship between time and the number of articles existed for papers focusing on mammals, fish, or invertebrates, as well as papers that were not taxon-specific (*R* ^2^ range: 0.001 – 0.15, *P* range: 0.26 – 0.92). Because data for reptile and amphibians were highly non-normal, I ran Spearman rank correlation tests on these data with similar results (*r* range: 0 – 0.43, *P* range: 0.21 – 1).

**Figure 2:**
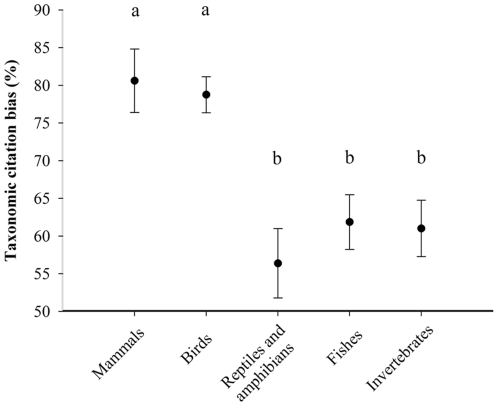
Taxonomic citation bias (% of citations on studied taxon) for articles from five major animal taxonomic groups (*n* = 15–20). Values are displayed as mean±s.e.m., and significant differences among groups are denoted by letters (a≠b). Data from reptiles and amphibians were pooled due to low sample sizes (reptiles: *n* = 11, amphibians: *n* = 5).

Taxonomic citation bias was significantly influenced by taxon (Fig. 2; *F*_4,90_ = 8.8, *P*<0.001). A significant relationship between taxonomic citation bias and the relative citation rate (total number of times an article has been cited ÷ the number of years since its publication) did not exist (*F*_1,93_ = 0.050, *R* ^2^<0.001, *P* = 0.82). Further, relative citation rate was not influenced by taxon. Compared to articles on animal behavior in general, the entire sample of parental care articles exhibits lower levels of taxonomic citation bias (81±3% *versus* 68±2%; *t*_131_ = 3.5, *P*<0.001). However, the endothermic taxa dominating parental care research (75% of articles) exhibited taxonomic citation bias similar to other areas of behavior (80±2%; *t*_76_ = 0.33, *P* = 0.75).

## Discussion

My results regarding the taxon-based composition of parental care research from 2001–2010 is in general agreement with [4], which showed that bias related to taxonomy was static from 1992–2000 in ecological and evolutionary research. However, the absolute number of papers on bird parental care significantly increased over the past decade in contrast to those from other taxonomic groups. Yet, the large proportion of articles on bird parental care does not necessarily equate to high levels of taxonomic bias in parental care research. For instance, research on birds may dominate the literature because the majority of parental care is exhibited by bird species (but see below).

Determining the exact degree to which taxonomic bias exists is difficult because the daunting task of exploring parental care across taxa is unfinished. Yet, evidence accumulated by researchers in combination with my results suggests significant taxonomic bias does persist in parental care research. For example, genus *Drosophila* is a member of an incredibly speciose family (Diptera, true flies: ∼240,000 species [9]) relative to vertebrates (∼58,000 species [10]), and they exhibit adaptive oviposition-site selection (reviewed in [11]). However, invertebrates represent less than 11% of parental care research (Fig. 1a) despite encompassing 95% of all animal species [10]. Even among vertebrates, birds comprise approximately two-thirds of parental care research (Fig. 1a) but just 20% of species. Although bony fish represent only 9% of parental care research (Fig. 1a), they comprise half of all vertebrate species, and parental care is exhibited by 20% of bony fish families [12]. Thus, non-avian species exhibiting parental care likely greatly outnumber birds, yet comprise only 42% of parental care research (Fig. 1a).

Parental care research on endothermic taxa also refers to a relatively narrow range of taxonomic groups when discussing its context (taxonomic citation bias: Fig. 2). This result agrees with [8], which found that research on mammal and bird behavior exhibited relatively high levels of taxonomic citation bias. However, there seems to be no selective advantage to taxonomic citation bias because parental care articles with relatively high bias were not cited more often than articles with less bias. This may be due, in part, to a dilution effect in which the likelihood a given article on bird parental care is cited will be reduced because it is in a large pool of similar articles.

Together, my results demonstrate that parental care research exhibits significant bias with regard to taxonomy. Specifically, research on birds likely represents a disproportionate amount of parental care research (Fig. 1a,b), and it refers to a relatively narrow range of taxonomic groups when discussing its context (Fig. 2). Notably, the levels of taxonomic and taxonomic citation bias in parental care research on birds did not decline over the past decade— in fact, taxonomic bias may have increased. However, in accordance with [4], my results should not be interpreted as evidence of an ‘ornithological Mafia’ conspiring to suppress other taxonomic groups. Rather, rational hypotheses can be generated by examining the growing body of literature on bias related to taxonomy [4], [5], [7], [8].

Birds may be over-represented in parental care research for several reasons. In accordance with the second tenet of parental care research, research on birds may dominate the literature because birds are particularly suitable parental care models. Thus, the ‘ideal model’ hypothesis predicts that, better than any other taxa, birds fulfill the following criteria of a particularly suitable model taxon: (1) tractability (easy to locate, obtain, and manipulate), and (2) generality (exhibit a biological phenomenon or trait of broad significance). Avian parental care systems demonstrate fairly conspicuous and quantifiable behaviors (e.g., frequency and duration of nest attendance), and they are also amenable to manipulation (e.g., clutch reduction or enlargement). Yet, non-avian parental care systems also offer these advantages. For example, developmental variables of widespread significance (temperature, hydration, respiration, and/or predation risk) can be easily manipulated in other taxa exhibiting nest-attending behaviors (fish: [13]; reptiles: [14], [15]). Also, birds typically display an atypical mode of parental care where both parents provide care [1]. Yet, female-only parental care (only females provide care) is the predominant mode of care by other internally fertilizing vertebrates (e.g., mammals, reptiles, and poeciliid fish). It is also most prevalent among internally fertilizing species within major taxa in which external fertilization predominates (e.g., fish and amphibians) [16]. Female-only care is also the most common parental care mode among terrestrial arthropods [17]. Thus, evidence fails to adequately support the ‘ideal model’ hypothesis for the over-representation of birds in parental care research.

Bias favoring birds in parental care research may simply reflect societal preferences. In fact, the ‘societal preference’ hypothesis is rooted in previous research. For example, Wilson and colleagues demonstrated positive relationships between the amount of taxon-specific scientific research output and the number of taxon-specific web pages [18]. Also, urban residents tend to like birds and larger mammals (e.g., dogs), dislike invertebrates and smaller mammals (e.g., rodents), and cite bird or mammal observation as primary motives for taking walks [19]. Although a person's taxonomic bias may be rational (e.g., phobia of invertebrates associated with contaminated food), it may also be largely emotional and due to his or her culture, age, or gender (reviewed in [19]). Thus, the general public may be more interested in bird parental care because it can identify more with a system in which both parents care for their offspring. In turn, this can influence researchers directly (e.g., as members of society, they too are more naturally interested in bird parental care) or indirectly (e.g., bird parental care research may be relatively highly valued by publicly funded granting agencies, which influences researchers to choose birds as a study taxon). Notably, this hypothesis may also provide a mechanism for taxonomic chauvinism across disciplines. However, further data is required to support or refute the ‘societal preference’ hypothesis.

Parental care research on birds may cite a significantly high proportion of papers focusing on birds for at least two reasons. The ‘density-dependent’ hypothesis states that taxonomic citation bias simply reflects the taxon-based composition of parental care research. For example, a researcher looking for parental care references has five-fold more papers on bird parental care to choose from than from any other taxon (Fig. 1). Although this hypothesis would certainly explain high levels of bias in bird research, it also suggests that citation choice is random rather than logical. In refutation, research on non-avian groups did not cite bird parental care research five-fold more than research on other taxa because citation bias was >50% for every taxonomic group (Fig. 2). Alternatively, the ‘scope-dependent’ hypothesis states that citation bias in birds reflects the degree to which parental care traits in birds are relatively specific to birds (e.g., bi-parental nest attendance). This hypothesis predicts researchers investigating bird parental care are logically choosing appropriate articles. Yet, it also infers that these researchers are working in study systems with very narrow foci. Thus, results from such investigation may provide limited insight into parental care in general.

Few would argue the virtues of bias related to taxonomy in parental care research (or any other type of research). The ability to get research papers published and the rate at which research papers are cited play a large role in scientists' acquisition of funding and career trajectories. On a more philosophical note, bias related to taxonomy likely reduces the rate at which scientists accumulate knowledge about the proximate and ultimate mechanisms of parental care. Unfortunately, it appears biologists continued to make the same mistakes this decade as the one prior with regard to biased publication practices ([4]; Figs. 1a,b and 2). However, I hope future work addresses the above hypotheses, and I urge biologists across disciplines to thoughtfully choose the organisms they study and the papers they cite.

## Supporting Information

Supporting Information S1 **Articles entailing parental care published in** ***Animal Behaviour*** **,** ***Behavioral Ecology*** **,** ***Behavioral Ecology and Sociobiology*** **,** ***Ethology*** **,** ***Hormones and Behavior*** **, and** ***Physiology & Behavior*** **from 2001 through 2010 (** ***n*** **= 712).**
(DOC)
