# Supplementary material for: Taxonomic Chauvinism Revisited: Insight from Parental Care Research
Source: PLoS One. 2011 Aug 31;6(8):e24192. doi: 10.1371/journal.pone.0024192 (PMC3164163; doi:10.1371/journal.pone.0024192)
Supplement: Supporting Information S1 — Articles entailing parental care published in Animal Behaviour , Behavioral Ecology , Behavioral Ecology and Sociobiology , Ethology , Hormones and Behavior , and Physiology & Behavior from 2001 through 2010 ( n = 712). (DOC) [file pone.0024192.s001.doc]

**Supporting Information S1**

1. Ackerman JT, Eadie JM (2003) Current versus future reproduction: an experimental test of parental investment decisions using nest desertion by mallards (Anas platyrhynchos). Behavioral Ecology and Sociobiology 54: 264-273.

2. Ackerman JT, Eadie JM, Yarris GS, Loughman DL, McLandress MR (2003) Cues for investment: nest desertion in response to partial clutch depredation in dabbling ducks. Animal Behaviour 66: 871-883.

3. Addison B, Kitaysky AS, Hipfner JM (2008) Sex allocation in a monomorphic seabird with a single-egg clutch: test of the environment, mate quality, and female condition hypotheses. Behavioral Ecology and Sociobiology 63: 135-141.

4. Agrawal AF, Combs N, Brodie ED (2005) Insights into the costs of complex maternal care behavior in the burrower bug (Sehirus cinctus). Behavioral Ecology and Sociobiology 57: 566-574.

5. Albrecht T, Klvana P (2004) Nest crypsis, reproductive value of a clutch and escape decisions in incubating female mallards Anas platyrhynchos. Ethology 110: 603-613.

6. Allen T, Clarke JA (2005) Social learning of food preferences by white-tailed ptarmigan chicks. Animal Behaviour 70: 305-310.

7. Almasi B, Roulin A, Jenni-Eiermann S, Jenni L (2008) Parental investment and its sensitivity to corticosterone is linked to melanin-based coloration in barn owls. Hormones and Behavior 54: 217-223.

8. Almond REA, Brown GR, Keverne EB (2005) The effect of lowering prolactin on the expression parental care in paternally experienced male common marmosets (callithrix jacchus). Hormones and Behavior 48: 33.

9. Almond REA, Brown GR, Keverne EB (2006) Suppression of prolactin does not reduce infant care by parentally experienced male common marmosets (Callithrix jacchus). Hormones and Behavior 49: 673-680.

10. Alvergne A, Faurie C, Raymond M (2008) Developmental plasticity of human reproductive development: Effects of early family environment in modern-day France. Physiology & Behavior 95: 625-632.

11. Alvergne A, Faurie C, Raymond M (2009) Father-offspring resemblance predicts paternal investment in humans. Animal Behaviour 78: 61-69.

12. Alvergne A, Faurie C, Raymond M (2009) Variation in testosterone levels and male reproductive effort: Insight from a polygynous human population. Hormones and Behavior 56: 491-497.

13. Amat JA, Masero JA (2004) How Kentish plovers, Charadrius alexandrinus, cope with heat stress during incubation. Behavioral Ecology and Sociobiology 56: 26-33.

14. Anderholm S, Marshall RC, van der Jeugd HP, Waldeck P, Larsson K, et al. (2009) Nest parasitism in the barnacle goose: evidence from protein fingerprinting and microsatellites. Animal Behaviour 78: 167-174.

15. Anderson MG, Brunton DH, Hauber ME (2010) Reliable Information Content and Ontogenetic Shift in Begging Calls of Grey Warbler Nestlings. Ethology 116: 357-365.

16. Anderson MG, Brunton DH, Hauber ME (2010) Species specificity of grey warbler begging solicitation and alarm calls revealed by nestling responses to playbacks. Animal Behaviour 79: 401-409.

17. Andersson M (2005) Evolution of classical polyandry: Three steps to female emancipation. Ethology 111: 1-23.

18. Aquiloni L, Gherardi F (2008) Extended mother-offspring relationships in crayfish: The return behaviour of juvenile Procambarus clarkii. Ethology 114: 946-954.

19. Ardia DR, Clotfelter ED (2007) Individual quality and age affect responses to an energetic constraint in a cavity-nesting bird. Behavioral Ecology 18: 259-266.

20. Arnold C, Taborsky B (2010) Social experience in early ontogeny has lasting effects on social skills in cooperatively breeding cichlids. Animal Behaviour 79: 621-630.

21. Arroyo BE, De Cornulier T, Bretagnolle V (2002) Parental investment and parent-offspring conflicts during the postfledging period in Montagu's harriers. Animal Behaviour 63: 235-244.

22. Arroyo B, Mougeot F, Bretagnolle V (2001) Colonial breeding and nest defence in Montagu's harrier (Circus pygargus). Behavioral Ecology and Sociobiology 50: 109-115.

23. Asoh K, Yoshikawa T (2001) Female nest defense in a coral-reef fish, Dascyllus albisella, with uniparental male care. Behavioral Ecology and Sociobiology 51: 8-16.

24. Aubret F, Bonnet X, Shine R, Maumelat S (2005) Energy expenditure for parental care may be trivial for brooding pythons, Python regius. Animal Behaviour 69: 1043-1053.

25. Aviles JM, Perez-Contreras T, Navarro C, Soler JJ (2009) Male spotless starlings adjust feeding effort based on egg spots revealing ectoparasite load. Animal Behaviour 78: 993-999.

26. Badyaev AV, Hill GE (2002) Paternal care as a conditional strategy: distinct reproductive tactics associated with elaboration of plumage ornamentation in the house finch. Behavioral Ecology 13: 591-597.

27. Balenger SL, Johnson LS, Brubaker JL, Ostlind E (2007) Parental effort in relation to structural plumage coloration in the mountain bluebird (Sialia currucoides). Ethology 113: 838-846.

28. Bales KL, Kramer KM, Lewis-Reese AD, Carter CS (2005) Effects of stress on parental care are sexually dimorphic in prairie voles. Hormones and Behavior 48: 37.

29. Bales KL, Kramer KM, Lewis-Reese AD, Carter CS (2006) Effects of stress on parental care are sexually dimorphic in prairie voles. Physiology & Behavior 87: 424-429.

30. Bandoli JH (2002) Brood defense and filial cannibalism in the spottail darter (Etheostoma squamiceps): The effects of parental status and prior experience. Behavioral Ecology and Sociobiology 51: 222-226.

31. Bandoli JH (2006) Male spottail darters (Etheostoma squamiceps) do not use chemical or positional cues to discriminate between sired and foster eggs. Behavioral Ecology and Sociobiology 59: 606-613.

32. Beaulieu M, Raclot T, Dervaux A, Le Maho Y, Ropert-Coudert Y, et al. (2009) Can a handicapped parent rely on its partner? An experimental study within Adelie penguin pairs. Animal Behaviour 78: 313-320.

33. Beaulieu M, Thierry AM, Raclot T, Maho Y, Ropert-Coudert Y, et al. (2009) Sex-specific parental strategies according to the sex of offspring in the Adelie penguin. Behavioral Ecology 20: 878-883.

34. Bender N, Heg-Bachar Z, Oliveira RF, Canario AVM, Taborsky M (2008) Hormonal control of brood care and social status in a cichlid fish with brood care helpers. Physiology & Behavior 94: 349-358.

35. Bester-Meredith JK, Marler CA (2003) The association between male offspring aggression and paternal and maternal behavior of Peromyscus mice. Ethology 109: 797-808.

36. Bester-Meredith JK, Marler CA (2007) Social experience during development and female offspring aggression in Peromyscus mice. Ethology 113: 889-900.

37. Bickford DP (2004) Differential parental care behaviors of arboreal and terrestrial microhylid frogs from Papua New Guinea. Behavioral Ecology and Sociobiology 55: 402-409.

38. Bijleveld AI, Mullers RHE (2009) Reproductive effort in biparental care: an experimental study in long-lived Cape gannets. Behavioral Ecology 20: 736-744.

39. Blackmer AL, Mauck RA, Ackerman JT, Huntington CE, Nevitt GA, et al. (2005) Exploring individual quality: basal metabolic rate and reproductive performance in storm-petrels. Behavioral Ecology 16: 906-913.

40. Blanchard P, Hanuise N, Dano S, Weimerskirch H (2007) Offspring sex ratio in relation to parental structural size and body condition in the long-lived wandering albatross (Diomedea exulans). Behavioral Ecology and Sociobiology 61: 767-773.

41. Bloch M, Peleg I, Koren D, Aner H, Klein E (2007) Long-term effects of early parental loss due to divorce on the HPA axis. Hormones and Behavior 51: 516-523.

42. Bohn KM, Moss CF, Wilkinson GS (2009) Pup guarding by greater spear-nosed bats. Behavioral Ecology and Sociobiology 63: 1693-1703.

43. Bohn KM, Wilkinson GS, Moss CF (2007) Discrimination of infant isolation calls by female greater spear-nosed bats, Phyllostomus hastatus. Animal Behaviour 73: 423-432.

44. Bonato M, Evans MR, Cherry MI (2009) Investment in eggs is influenced by male coloration in the ostrich, Struthio camelus. Animal Behaviour 77: 1027-1032.

45. Boncoraglio G, Martinelli R, Saino N (2008) Sex-related asymmetry in competitive ability of sexually monomorphic barn swallow nestlings. Behavioral Ecology and Sociobiology 62: 729-738.

46. Boncoraglio G, Rubolini D, Romano M, Martinelli R, Saino N (2006) Effects of elevated yolk androgens on perinatal begging behavior in yellow-legged gull (Larus michahellis) chicks. Hormones and Behavior 50: 442-447.

47. Boncoraglio G, Saino N, Garamszegi LZ (2009) Begging and cowbirds: brood parasites make hosts scream louder. Behavioral Ecology 20: 215-221.

48. Bonnevier K, Lindstrom K, Mary CS (2003) Parental care and mate attraction in the Florida flagfish, Jordanella floridae. Behavioral Ecology and Sociobiology 53: 358-363.

49. Bosch J, Vicens N (2005) Sex allocation in the solitary bee Osmia cornuta: do females behave in agreement with Fisher's theory? Behavioral Ecology and Sociobiology 59: 124-132.

50. Bosch J, Vicens N (2006) Relationship between body size, provisioning rate, longevity and reproductive success in females of the solitary bee Osmia cornuta. Behavioral Ecology and Sociobiology 60: 26-33.

51. Boulton RL, Richard Y, Armstrong DP (2010) The Effect of Male Incubation Feeding, Food and Temperature on the Incubation Behaviour of New Zealand Robins. Ethology 116: 490-497.

52. Bouwman KM, Lessells CM, Komdeur J (2005) Male reed buntings do not adjust parental effort in relation to extrapair paternity. Behavioral Ecology 16: 499-506.

53. Branchi I, D'Andrea I, Gracci F, Santucci D, Alleva E (2009) Birth spacing in the mouse communal nest shapes adult emotional and social behavior. Physiology & Behavior 96: 532-539.

54. Bredy TW, Lee AW, Meaney MJ, Brown RE (2004) Effect of neonatal handling and paternal care on offspring cognitive development in the monogamous California mouse (Peromyscus californicus). Hormones and Behavior 46: 30-38.

55. Brooks PL, Vella ET, Wynne-Edwards KE (2005) Dopamine agonist treatment before and after the birth reduces prolactin concentration but does not impair paternal responsiveness in Djungarian hamsters Phodopus campbelli. Hormones and Behavior 47: 358-366.

56. Broom M, Ruxton GD (2002) A game theoretical approach to conspecific brood parasitism. Behavioral Ecology 13: 321-327.

57. Brotherton PNM, Clutton-Brock TH, O'Riain MJ, Gaynor D, Sharpe L, et al. (2001) Offspring food allocation by parents and helpers in a cooperative mammal. Behavioral Ecology 12: 590-599.

58. Brown GR (2001) Sex-biased investment in nonhuman primates: can Trivers & Willard's theory be tested. Animal Behaviour 61: 683-694.

59. Brumm H, Zollinger SA, Slater PJB (2009) Developmental stress affects song learning but not song complexity and vocal amplitude in zebra finches. Behavioral Ecology and Sociobiology 63: 1387-1395.

60. Buchanan KL, Goldsmith AR, Hinde CA, Griffith SC, Kilner RM (2007) Does testosterone mediate the trade-off between nestling begging and growth in the canary (Serinus canaria)? Hormones and Behavior 52: 664-671.

61. Budden AE, Beissinger SR (2009) Resource allocation varies with parental sex and brood size in the asynchronously hatching green-rumped parrotlet (Forpus passerinus). Behavioral Ecology and Sociobiology 63: 637-647.

62. Budden AE, Wright J (2005) Learning during competitive positioning in the nest: do nestlings use ideal free 'foraging' tactics? Behavioral Ecology and Sociobiology 58: 227-236.

63. Bussiere LF, Basit HA, Gwynne DT (2005) Preferred males are not always good providers: female choice and male investment in tree crickets. Behavioral Ecology 16: 223-231.

64. Butchart SHM, Kilner RM, Fuisz T, Davies NB (2003) Differences in the nestling begging calls of hosts and host-races of the common cuckoo, Cuculus canorus. Animal Behaviour 65: 345-354.

65. Calatayud F, Belzung C (2001) Emotional reactivity in mice, a case of nongenetic heredity? Physiology & Behavior 74: 355-362.

66. Cameron NM, Fish EW, Meaney MJ (2008) Maternal influences on the sexual behavior and reproductive success of the female rat. Hormones and Behavior 54: 178-184.

67. Cameron-MacMillan ML, Walsh CJ, Wilhelm SI, Storey AE (2007) Male chicks are more costly to rear than females in a monogamous seabird, the Common Murre. Behavioral Ecology 18: 81-85.

68. Campbell JC, Laugero KD, Van Westerhuyzen JA, Hostetler CM, Cohen JD, et al. (2009) Costs of pair-bonding and paternal care in male prairie voles (Microtus ochrogaster). Physiology & Behavior 98: 367-373.

69. Campobello D, Sealy SG (2010) Enemy Recognition of Reed Warblers (Acrocephalus scirpaceus): Threats and Reproductive Value Act Independently in Nest Defence Modulation. Ethology 116: 498-508.

70. Canestrari D, Marcos JM, Baglione V (2005) Effect of parentage and relatedness on the individual contribution to cooperative chick care in carrion crows Corvus corone corone. Behavioral Ecology and Sociobiology 57: 422-428.

71. Cant MA (2006) A tale of two theories: parent-offspring conflict and reproductive skew. Animal Behaviour 71: 255-263.

72. Carranza J (2004) Sex allocation within broods: the intrabrood sharing-out hypothesis. Behavioral Ecology 15: 223-232.

73. Carranza J, Polo V, Valencia J, Mateos C, de la Cruz C (2008) How should breeders react when aided by helpers? Animal Behaviour 75: 1535-1542.

74. Carrillo J, Aparicio JM (2001) Nest defence behaviour of the Eurasian kestrel (Falco tinnunculus) against human predators. Ethology 107: 865-875.

75. Catry P, Phillips RA, Forcada J, Croxall JP (2006) Factors affecting the solution of a parental dilemma in albatrosses: at what age should chicks be left unattended? Animal Behaviour 72: 383-391.

76. Chalfoun AD, Martin TE (2007) Latitudinal variation in avian incubation attentiveness and a test of the food limitation hypothesis. Animal Behaviour 73: 579-585.

77. Charrier I, Mathevon N, Jouventin P (2003) Vocal signature recognition of mothers by fur seal pups. Animal Behaviour 65: 543-550.

78. Charrier I, Mathevon N, Jouventin P, Aubin T (2001) Acoustic communication in a black-headed gull colony: How do chicks identify their parents? Ethology 107: 961-974.

79. Chastel O, Lacroix A, Weimerskirch H, Gabrielsen GW (2005) Modulation of prolactin but not corticosterone responses to stress in relation to parental effort in a long-lived bird. Hormones and Behavior 47: 459-466.

80. Cheney KL, Cote IM (2003) The ultimate effect of being cleaned: does ectoparasite removal have reproductive consequences for damselfish clients? Behavioral Ecology 14: 892-896.

81. Chiver I, Morton ES, Stutchbury BJM (2007) Incubation delays territory defence by male blue-headed vireos, Vireo solitarius. Animal Behaviour 73: 143-148.

82. Chuang-Dobbs HC, Webster MS, Holmes RT (2001) Paternity and parental care in the black-throated blue warbler, Dendroica caerulescens. Animal Behaviour 62: 83-92.

83. Clark JA, Boersma PD, Olmsted DM (2006) Name that tune: call discrimination and individual recognition in Magellanic penguins. Animal Behaviour 72: 1141-1148.

84. Clark MM, Johnson J, Galef BG (2004) Sexual motivation suppresses paternal behaviour of male gerbils during their mates ' postpartum oestrus. Animal Behaviour 67: 49-57.

85. Clark MM, Moghaddas M, Galef BG (2002) Age at first mating affects parental effort and fecundity of female Mongolian gerbils. Animal Behaviour 63: 1129-1134.

86. Clark MM, Whiskin EE, Galef BG (2003) Mongolian gerbil fathers avoid newborn male pups, but not newborn female pups: olfactory control of early paternal behaviour. Animal Behaviour 66: 441-447.

87. Clotfelter ED, Chandler CR, Nolan V, Ketterson ED (2007) The influence of exogenous testosterone on the dynamics of nestling provisioning in dark-eyed juncos. Ethology 113: 18-25.

88. Clotfelter ED, Curren LJ, Murphy CE (2006) Mate choice and spawning success in the fighting fish Betta splendens: The importance of body size, display behavior and nest size. Ethology 112: 1170-1178.

89. Clotfelter ED, Nolan V, Ketterson ED (2001) The effects of experimentally elevated testosterone and food deprivation on food consumption and prey size preferences in male dark-eyed juncos (Junco hyemalis, Emberizidae : Passeriformes). Ethology 107: 439-449.

90. Clotfelter ED, O'Neal DM, Gaudioso JM, Casto JM, Parker-Renga IM, et al. (2004) Consequences of elevating plasma testosterone in females of a socially monogamous songbird: evidence of constraints on male evolution? Hormones and Behavior 46: 171-178.

91. Clutton-Brock TH, Russell AF, Sharpe LL (2004) Behavioural tactics of breeders in cooperative meerkats. Animal Behaviour 68: 1029-1040.

92. Cocroft RB (2002) Antipredator defense as a limited resource: unequal predation risk in broods of an insect with maternal care. Behavioral Ecology 13: 125-133.

93. Cook MI, Beissinger SR, Toranzos GA, Rodriguez RA, Arendt WJ (2005) Microbial infection affects egg viability and incubation behavior in a tropical passerine. Behavioral Ecology 16: 30-36.

94. Corbel H, Morlon F, Geiger S, Groscolas R (2009) State-dependent decisions during the fledging process of king penguin chicks. Animal Behaviour 78: 829-838.

95. Creighton JC (2005) Population density, body size, and phenotypic plasticity of brood size in a burying beetle. Behavioral Ecology 16: 1031-1036.

96. Cresswell W, Holt S, Reid JM, Whitfield DP, Mellanby RJ (2003) Do energetic demands constrain incubation scheduling in a biparental species? Behavioral Ecology 14: 97-102.

97. Crook TC, Flatt T, Smiseth PT (2008) Hormonal modulation of larval begging and growth in the burying beetle Nicrophorus vespilloides. Animal Behaviour 75: 71-77.

98. Cunningham EJA, Lewis S (2006) Parasitism of maternal investment selects for increased clutch size and brood reduction in a host. Behavioral Ecology 17: 126-131.

99. Curley JP, Champagne FA, Bateson P, Keverne EB (2008) Transgenerational effects of impaired maternal care on behaviour of offspring and grandoffspring. Animal Behaviour 75: 1551-1561.

100. D'Alba L, Shawkey MD, Korsten P, Vedder O, Kingma SA, et al. (2010) Differential deposition of antimicrobial proteins in blue tit (Cyanistes caeruleus) clutches by laying order and male attractiveness. Behavioral Ecology and Sociobiology 64: 1037-1045.

101. D'Amato FR, Rizzi R, Moles A (2006) Aggression and anxiety in pregnant mice are modulated by offspring characteristics. Animal Behaviour 72: 773-780.

102. Dawson RD, Bortolotti GR (2002) Experimental evidence for food limitation and sex-specific strategies of American kestrels (Falco sparverius) provisioning offspring. Behavioral Ecology and Sociobiology 52: 43-52.

103. de Ayala RM, Saino N, Moller AP, Anselmi C (2007) Mouth coloration of nestlings covaries with offspring quality and influences parental feeding behavior. Behavioral Ecology 18: 526-534.

104. de Jong TR, Chauke M, Harris BN, Saltzman W (2009) From here to paternity: Neural correlates of the onset of paternal behavior in California mice (Peromyscus californicus). Hormones and Behavior 56: 220-231.

105. de la Cruz C, Solis E, Valencia J, Chastel O, Sorci G (2003) Testosterone and helping behavior in the azure-winged magpie (Cyanopica cyanus): natural covariation and an experimental test. Behavioral Ecology and Sociobiology 55: 103-111.

106. De Neve L, Soler JJ (2002) Nest-building activity and laying date influence female reproductive investment in magpies: an experimental study. Animal Behaviour 63: 975-980.

107. De Neve L, Soler JJ, Soler M, Perez-Contreras T (2004) Nest size predicts the effect of food supplementation to magpie nestlings on their immunocompetence: an experimental test of nest size indicating parental ability. Behavioral Ecology 15: 1031-1036.

108. Dearborn DC (2001) Body condition and retaliation in the parental effort decisions of incubating great frigatebirds (Fregata minor). Behavioral Ecology 12: 200-206.

109. Dearborn DC, MacDade LS, Robinson S, Fink ADD, Fink ML (2009) Offspring development mode and the evolution of brood parasitism. Behavioral Ecology 20: 517-524.

110. DeMory ML, Thompson CF, Sakaluk SK (2010) Male quality influences male provisioning in house wrens independent of attractiveness. Behavioral Ecology 21: 1156-1164.

111. Desiardins JK, Stiver KA, Fitzpatrick JL, Balshine S (2008) Differential responses to territory intrusions in cooperatively breeding fish. Animal Behaviour 75: 595-604.

112. Desjardins JK, Stiver KA, Fitzpatrick JL, Milligan N, Van Der Kraak GJ, et al. (2008) Sex and status in a cooperative breeding fish: behavior and androgens. Behavioral Ecology and Sociobiology 62: 785-794.

113. Dey CJ, O'Connor CM, Gilmour KM, Van Der Kraak G, Cooke SJ (2010) Behavioral and physiological responses of a wild teleost fish to cortisol and androgen manipulation during parental care. Hormones and Behavior 58: 599-605.

114. Dias RI, Castilho L, Macedo RH (2010) Experimental Evidence that Sexual Displays are Costly for Nest Survival. Ethology 116: 1011-1019.

115. Dick JTA, Bailey RJE, Elwood RW (2002) Maternal care in the rockpool amphipod Apherusa jurinei: developmental and environmental cues. Animal Behaviour 63: 707-713.

116. Dickens M, Berridge D, Hartley IR (2008) Biparental care and offspring begging strategies: hungry nestling blue tits move towards the father. Animal Behaviour 75: 167-174.

117. Dickens M, Hartley IR (2007) Differences in parental food allocation rules: evidence for sexual conflict in the blue tit? Behavioral Ecology 18: 674-679.

118. Dickinson JL (2003) Male share of provisioning is not influenced by actual or apparent loss of paternity in western bluebirds. Behavioral Ecology 14: 360-366.

119. Dickinson JL (2004) Facultative sex ratio adjustment by western bluebird mothers with stay-at-home helpers-at-the-nest. Animal Behaviour 68: 373-380.

120. Dickinson JL, Euaparadorn M, Greenwald K, Mitra C, Shizuka D (2009) Cooperation and competition: nepotistic tolerance and intrasexual aggression in western bluebird winter groups. Animal Behaviour 77: 867-872.

121. Dobbs RC, Styrsky JD, Thompson CF (2006) Clutch size and the costs of incubation in the house wren. Behavioral Ecology 17: 849-856.

122. Dobler R, Kolliker M (2009) Behavioural attainability of evolutionarily stable strategies in repeated interactions. Animal Behaviour 77: 1427-1434.

123. Dolan AC, Murphy MT, Redmond LJ, Duffield D (2009) Maternal characteristics and the production and recruitment of sons in the eastern kingbird (Tyrannus tyrannus). Behavioral Ecology and Sociobiology 63: 1527-1537.

124. Doody LM, Wilhelm SI, McKay DW, Walsh CJ, Storey AE (2008) The effects of variable foraging conditions on common murre (Uria aalge) corticosterone concentrations and parental provisioning. Hormones and Behavior 53: 140-148.

125. Dor R, Kedar H, Winkler DW, Lotem A (2007) Begging in the absence of parents: a "quick on the trigger" strategy to minimize costly misses. Behavioral Ecology 18: 97-102.

126. Draganoiu TI, Nagle L, Musseau R, Kreutzer M (2006) In a songbird, the black redstart, parents use acoustic cues to discriminate between their different fledglings. Animal Behaviour 71: 1039-1046.

127. Drake A, Fraser D, Weary DM (2008) Parent-offspring resource allocation in domestic pigs. Behavioral Ecology and Sociobiology 62: 309-319.

128. Drickamer LC, Robinson AS, Mossman CA (2001) Differential responses to same and opposite sex odors by adult house mice are associated with anogenital distance. Ethology 107: 509-519.

129. Drummond H, Rodriguez C (2009) No reduction in aggression after loss of a broodmate: a test of the brood size hypothesis. Behavioral Ecology and Sociobiology 63: 321-327.

130. Drummond H, Torres R, Juarez CR, Kim SY (2010) Is kin cooperation going on undetected in marine bird colonies? Behavioral Ecology and Sociobiology 64: 647-655.

131. Duckworth RA (2006) Behavioral correlations across breeding contexts provide a mechanism for a cost of aggression. Behavioral Ecology 17: 1011-1019.

132. Duckworth RA, Badyaev AV, Parlow AF (2003) Elaborately ornamented males avoid costly parental care in the house finch (Carpodacus mexicanus): a proximate perspective. Behavioral Ecology and Sociobiology 55: 176-183.

133. Dugas MB (2009) House sparrow, Passer domesticus, parents preferentially feed nestlings with mouth colours that appear carotenoid-rich. Animal Behaviour 78: 767-772.

134. Dugas MB, Rosenthal GG (2010) Carotenoid-rich mouth colors influence the conspicuousness of nestling birds. Behavioral Ecology and Sociobiology 64: 455-462.

135. Dugdale HL, Ellwood SA, Macdonald DW (2010) Alloparental behaviour and long-term costs of mothers tolerating other members of the group in a plurally breeding mammal. Animal Behaviour 80: 721-735.

136. Ebensperger LA, Ramirez-Otarola N, Leon C, Ortiz ME, Croxatto HB (2010) Early fitness consequences and hormonal correlates of parental behaviour in the social rodent, Octodon degus. Physiology & Behavior 101: 509-517.

137. Ebenspherger LA, Cofre H (2001) On the evolution of group-living in the New World cursorial hystricognath rodents. Behavioral Ecology 12: 227-236.

138. Eggers S, Griesser M, Ekman J (2005) Predator-induced plasticity in nest visitation rates in the Siberian jay (Perisoreus infaustus). Behavioral Ecology 16: 309-315.

139. Eggers S, Griesser M, Ekman J (2008) Predator-induced reductions in nest visitation rates are modified by forest cover and food availability. Behavioral Ecology 19: 1056-1062.

140. Eikenaar C, Richardson DS, Brouwer L, Komdeur J (2007) Parent presence, delayed dispersal, and territory acquisition in the Seychelles warbler. Behavioral Ecology 18: 874-879.

141. Eising CM, Groothuis TGG (2003) Yolk androgens and begging behaviour in black-headed gull chicks: an experimental field study. Animal Behaviour 66: 1027-1034.

142. Eldegard K, Sonerud GA (2010) Experimental increase in food supply influences the outcome of within-family conflicts in Tengmalm's owl. Behavioral Ecology and Sociobiology 64: 815-826.

143. Elliott KH, Gaston AJ, Crump D (2010) Sex-specific behavior by a monomorphic seabird represents risk partitioning. Behavioral Ecology 21: 1024-1032.

144. Emlen ST, Wrege PH (2004) Division of labour in parental care behaviour of a sex-role-reversed shorebird, the wattled jacana. Animal Behaviour 68: 847-855.

145. Fargallo JA, De Leon A, Potti J (2001) Nest-maintenance effort and health status in chinstrap penguins, Pygoscelis antarctica: the functional significance of stone-provisioning behaviour. Behavioral Ecology and Sociobiology 50: 141-150.

146. Fashing PJ (2001) Male and female strategies during intergroup encounters in guerezas (Colobus guereza): evidence for resource defense mediated through males and a comparison with other primates. Behavioral Ecology and Sociobiology 50: 219-230.

147. Fedy BC, Martin TE (2009) Male songbirds provide indirect parental care by guarding females during incubation. Behavioral Ecology 20: 1034-1038.

148. Fey K, Trillmich F (2008) Sibling competition in guinea pigs (Cavia aperea f. porcellus): scrambling for mother's teats is stressful. Behavioral Ecology and Sociobiology 62: 321-329.

149. Field J (2005) The evolution of progressive provisioning. Behavioral Ecology 16: 770-778.

150. Fiorini VD, Tuero DT, Reboreda JC (2009) Shiny cowbirds synchronize parasitism with host laying and puncture host eggs according to host characteristics. Animal Behaviour 77: 561-568.

151. Fite JE, French JA, Patera KJ, Hopkins EC, Rukstalis M, et al. (2005) Elevated urinary testosterone excretion and decreased maternal caregiving effort in marmosets when conception occurs during the period of infant dependence. Hormones and Behavior 47: 39-48.

152. Fleming AS, Corter C, Stallings J, Steiner M (2002) Testosterone and prolactin are associated with emotional responses to infant cries in new fathers. Hormones and Behavior 42: 399-413.

153. Forester DC, Cameron M, Forester JD (2008) Nest and egg recognition by salamanders in the genus Desmognathus: A comprehensive re-examination. Ethology 114: 965-976.

154. Foster VS, Burley NT (2007) Sex allocation in response to maternal condition: Different tactics of care-giving by male and female zebra finches. Ethology 113: 511-520.

155. Frazier CRM, Trainor BC, Cravens CJ, Whitney TK, Marler CA (2006) Paternal behavior influences development of aggression and vasopressin expression in male California mouse offspring. Hormones and Behavior 50: 699-707.

156. Garcia-Gonzalez F, Gomendio M (2003) A field test of the intraspecific brood parasitism hypothesis in the golden egg bug (Phyllomorpha laciniata). Behavioral Ecology and Sociobiology 53: 332-339.

157. Garcia-Gonzalez F, Gomendio M (2003) Oviposition site selection and oviposition stimulation by conspecifics in the golden egg bug (Phyllomorpha laciniata): implications for female fitness. Behavioral Ecology and Sociobiology 53: 385-392.

158. Garcia-Navas V, Sanz JJ (2010) Flexibility in the Foraging Behavior of Blue Tits in Response to Short-Term Manipulations of Brood Size. Ethology 116: 744-754.

159. Garcia-Pena GE, Thomas GH, Reynolds JD, Szekely T (2009) Breeding systems, climate, and the evolution of migration in shorebirds. Behavioral Ecology 20: 1026-1033.

160. Garcia-Vigon E, Veiga JP, Cordero PJ (2009) Male feeding rate and extrapair paternity in the facultatively polygynous spotless starling. Animal Behaviour 78: 1335-1341.

161. Gardner JL, Magrath RD, Olsen PD (2004) Speckled warblers break cooperative rules: absence of helping in a group-living member of the Pardalotidae. Animal Behaviour 67: 719-728.

162. Geffen E, Yom-Tov Y (2001) Factors affecting the rates of intraspecific nest parasitism among Anseriformes and Galliformes. Animal Behaviour 62: 1027-1038.

163. Ghalambor CK, Martin TE (2002) Comparative manipulation of predation risk in incubating birds reveals variability in the plasticity of responses. Behavioral Ecology 13: 101-108.

164. Gil D, Marzal A, de Lope F, Puerta M, Moller AP (2006) Female house martins (Delichon urbica) reduce egg androgen deposition in response to a challenge of their immune system. Behavioral Ecology and Sociobiology 60: 96-100.

165. Gilchrist JS, Russell AF (2007) Who cares? Individual contributions to pup care by breeders vs non-breeders in the cooperatively breeding banded mongoose (Mungos mungo). Behavioral Ecology and Sociobiology 61: 1053-1060.

166. Gill SA, Costa LM, Hau M (2008) Males of a single-brooded tropical bird species do not show increases in testosterone during social challenges. Hormones and Behavior 54: 115-124.

167. Gill SA, Neudorf DLH, Sealy SG (2008) Do hosts discriminate between sexually dichromatic male and female brown-headed cowbirds? Ethology 114: 548-556.

168. Ginther AJ, Snowdon CT (2009) Expectant parents groom adult sons according to previous alloparenting in a biparental cooperatively breeding primate. Animal Behaviour 78: 287-297.

169. Glassey B, Forbes S (2002) Muting individual nestlings reduces parental foraging for the brood. Animal Behaviour 63: 779-786.

170. Glassey B, Forbes S (2003) Why brown-headed cowbirds do not influence red-winged blackbird parent behaviour. Animal Behaviour 65: 1235-1246.

171. Gomendio M, Garcia-Gonzalez F, Reguera P, Rivero A (2008) Male egg carrying in Phyllomorpha laciniata is favoured by natural not sexual selection. Animal Behaviour 75: 763-770.

172. Gomendio M, Reguera P (2001) Egg carrying in the golden egg bug (Phyllomorpha laciniata): parental care, parasitism, or both? Reply to Kaitala et al. Behavioral Ecology 12: 369-373.

173. Gomez R, Oliveira R, Leal I (2005) Psychoendocrinology of human parental behaviour - Changes in testosterone, estradiol and cortisol in response to infant versus control stimuli. Hormones and Behavior 48: 107.

174. Gonzalez-Solis J (2004) Regulation of incubation shifts near hatching by giant petrels: a timed mechanism, embryonic signalling or food availability? Animal Behaviour 67: 663-671.

175. Good TC, Harris KK, Ihunnah CA (2005) Corticosteroids as potential mechanism regulating variability in reproductive success in monogamous oldfield mice (Peromyscus polionotus). Physiology & Behavior 86: 96-102.

176. Goodship NM, Buchanan KL (2007) Nestling testosterone controls begging behaviour in the pied flycatcher, Ficedula hypoleuca. Hormones and Behavior 52: 454-460.

177. Gordon I, Zagoory-Sharon O, Leckman JF, Feldman R (2010) Oxytocin, cortisol, and triadic family interactions. Physiology & Behavior 101: 679-684.

178. Gordon I, Zagoory-Sharon O, Leckman JF, Feldman R (2010) Prolactin, Oxytocin, and the development of paternal behavior across the first six months of fatherhood. Hormones and Behavior 58: 513-518.

179. Gorman HE, Nager RG (2003) State-dependent incubation behaviour in the zebra finch. Animal Behaviour 65: 745-754.

180. Goubault M, Scott D, Hardy ICW (2007) The importance of offspring value: maternal defence in parasitoid contests. Animal Behaviour 74: 437-446.

181. Goymann W, Wingfield JC (2004) Competing females and caring males. Sex steroids in African black coucals, Centropus grillii. Animal Behaviour 68: 733-740.

182. Goymann W, Wittenzellner A, Wingfield JC (2004) Competing females and caring males. Polyandry and sex-role reversal in African black coucals, Centropus grillii. Ethology 110: 807-823.

183. Grans A, Altimiras J (2007) Ontogeny of vocalizations and movements in response to cooling in chickens fetuses. Physiology & Behavior 91: 229-239.

184. Gravel MA, Cooke SJ (2009) Influence of Inter-Lake Variation in Natural Nest Predation Pressure on the Parental Care Behaviour of Smallmouth Bass (Micropterus dolomieu). Ethology 115: 608-616.

185. Gray CM, Hamer KC (2001) Prefledging mass recession in Manx shearwaters: parental desertion or nestling anorexia? Animal Behaviour 62: 705-709.

186. Green BS, McCormick MI (2005) O-2 replenishment to fish nests: males adjust brood care to ambient conditions and brood development. Behavioral Ecology 16: 389-397.

187. Green DJ (2002) Pair bond duration influences paternal provisioning and the primary sex ratio of brown thornbill broods. Animal Behaviour 64: 791-800.

188. Grieco F (2001) Short-term regulation of food-provisioning rate and effect on prey size in blue tits, Parus caeruleus. Animal Behaviour 62: 107-116.

189. Grieco F (2002) Time constraint on food choice in provisioning blue tits, Parus caeruleus: the relationship between feeding rate and prey size. Animal Behaviour 64: 517-526.

190. Griesser M (2003) Nepotistic vigilance behavior in Siberian jay parents. Behavioral Ecology 14: 246-250.

191. Griesser M, Ekman A (2004) Nepotistic alarm calling in the Siberian jay, Perisoreus infaustus. Animal Behaviour 67: 933-939.

192. Griesser M, Ekman J (2005) Nepotistic mobbing behaviour in the Siberian jay, Perisoreus infaustus. Animal Behaviour 69: 345-352.

193. Griffith SC, Lyon BE, Montgomerie R (2004) Quasi-parasitism in birds. Behavioral Ecology and Sociobiology 56: 191-200.

194. Griggio M, Matessi G, Pilastro A (2003) Male rock sparrow (Petronia petronia) nest defence correlates with female ornament size. Ethology 109: 659-669.

195. Griggio M, Matessi G, Pilastro A (2005) Should I stay or should I go? Female brood desertion and male counterstrategy in rock sparrows. Behavioral Ecology 16: 435-441.

196. Griggio M, Pilastro A (2007) Sexual conflict over parental care in a species with female and male brood desertion. Animal Behaviour 74: 779-785.

197. Grim T (2006) Cuckoo growth performance in parasitized and unused hosts: not only host size matters. Behavioral Ecology and Sociobiology 60: 716-723.

198. Grim T, Honza M (2001) Does supernormal stimulus influence parental behaviour of the cuckoo's host? Behavioral Ecology and Sociobiology 49: 322-329.

199. Grim T, Rutila J, Cassey P, Hauber ME (2009) The cost of virulence: an experimental study of egg eviction by brood parasitic chicks. Behavioral Ecology 20: 1138-1146.

200. Grindstaff JL, Buerkle CA, Casto JM, Nolan V, Ketterson ED (2001) Offspring sex ratio is unrelated to male attractiveness in dark-eyed juncos (Junco hyemalis). Behavioral Ecology and Sociobiology 50: 312-316.

201. Grodzinski U, Erev I, Lotem A (2008) Can hungry nestlings be trained to reduce their begging? Behavioral Ecology 19: 116-125.

202. Groscolas R, Lacroix A, Robin JP (2008) Spontaneous egg or chick abandonment in energy-depleted king penguins: A role for corticosterone and prolactin? Hormones and Behavior 53: 51-60.

203. Gruebler MU, Naef-Daenzer B (2008) Postfledging parental effort in barn swallows: evidence for a trade-off in the allocation of time between broods. Animal Behaviour 75: 1877-1884.

204. Gruebler MU, Naef-Daenzer B (2010) Brood overlap and male ornamentation in the double-brooded barn swallow. Behavioral Ecology 21: 513-519.

205. Gruter C, Taborsky B (2004) Mouthbrooding and biparental care: an unexpected combination, but male brood care pays. Animal Behaviour 68: 1283-1289.

206. Gruter C, Taborsky B (2005) Sex ratio and the sexual conflict about brood care in a biparental mouthbrooder. Behavioral Ecology and Sociobiology 58: 44-52.

207. Hager R, Johnstone RA (2007) Maternal and offspring effects influence provisioning to mixed litters of own and alien young in mice. Animal Behaviour 74: 1039-1045.

208. Hahn S, Bauer S (2008) Dominance in feeding territories relates to foraging success and offspring growth in brown skuas Catharacta antarctica lonnbergi. Behavioral Ecology and Sociobiology 62: 1149-1157.

209. Hale RE (2008) Evidence that context-dependent mate choice for parental care mirrors bene fits to offspring. Animal Behaviour 75: 1283-1290.

210. Hale RE, St Mary CM (2007) Nest tending increases reproductive success, sometimes: environmental effects on paternal care and mate choice in flagfish. Animal Behaviour 74: 577-588.

211. Hamer KC, Quillfeldt P, Masello JF, Fletcher KL (2006) Sex differences in provisioning rules: responses of Manx shearwaters to supplementary chick feeding. Behavioral Ecology 17: 132-137.

212. Hansen BT, Slagsvold T (2004) Early learning affects social dominance: interspecifically cross-fostered tits become subdominant. Behavioral Ecology 15: 262-268.

213. Hanson KC, Abizaid A, Cooke SJ (2009) Causes and consequences of voluntary anorexia during the parental care period of wild male smallmouth bass (Micropterus dolomieu). Hormones and Behavior 56: 503-509.

214. Hanson KC, O'Connor CM, Van Der Kraak G, Cooke SJ (2009) Paternal aggression towards a brood predator during parental care in wild smallmouth bass is not correlated with circulating testosterone and cortisol concentrations. Hormones and Behavior 55: 495-499.

215. Harding AMA, Kitaysky AS, Hamer KC, Hall ME, Welcker J, et al. (2009) Impacts of experimentally increased foraging effort on the family: offspring sex matters. Animal Behaviour 78: 321-328.

216. Hardling R, Kaitala A (2001) Conflict of interest between sexes over cooperation: a supergame on egg carrying and mating in a coreid bug. Behavioral Ecology 12: 659-665.

217. Hardling R, Kaitala AJ (2004) Male brood care without paternity increases mating success. Behavioral Ecology 15: 715-721.

218. Hargitai R, Arnold KE, Herenyi M, Prechl J, Torok J (2009) Egg composition in relation to social environment and maternal physiological condition in the collared flycatcher. Behavioral Ecology and Sociobiology 63: 869-882.

219. Hauber ME (2002) Is reduced clutch size a cost of parental care in Eastern Phoebes (Sayornis phoebe)? Behavioral Ecology and Sociobiology 51: 503-509.

220. Hauber ME (2003) Hatching asynchrony, nestling competition, and the cost of interspecific brood parasitism. Behavioral Ecology 14: 227-235.

221. Hauber ME, Kilner RM (2007) Coevolution, communication, and host chick mimicry in parasitic finches: who mimics whom? Behavioral Ecology and Sociobiology 61: 497-503.

222. Hauber ME, Moskat C (2008) Shared parental care is costly for nestlings of common cuckoos and their great reed warbler hosts. Behavioral Ecology 19: 79-86.

223. Hayes LD, Solomon NG (2006) Mechanisms of maternal investment by communal prairie voles, Microtus ochrogaster. Animal Behaviour 72: 1069-1080.

224. Hayes UL, De Vries GJ (2007) Role of pregnancy and parturition in induction of maternal behavior in prairie voles (Microtus ochrogaster). Hormones and Behavior 51: 265-272.

225. Heeb P, Schwander T, Faoro S (2003) Nestling detectability affects parental feeding preferences in a cavity-nesting bird. Animal Behaviour 66: 637-642.

226. Heg D, Heyl S, Rasa OAE, Peschke K (2006) Reproductive skew and communal breeding in the subsocial beetle Parastizopus armaticeps. Animal Behaviour 71: 427-437.

227. Hegyi G, Garamszegi LZ, Eens M (2008) The roles of ecological factors and sexual selection in the evolution of white wing patches in ducks. Behavioral Ecology 19: 1208-1216.

228. Helfenstein F, Berthouly A, Tanner M, Karadas F, Richner H (2008) Nestling begging intensity and parental effort in relation to prelaying carotenoid availability. Behavioral Ecology 19: 108-115.

229. Heying HE (2001) Social and reproductive behaviour in the Madagascan poison frog, Mantella laevigata, with comparisons to the dendrobatids. Animal Behaviour 61: 567-577.

230. Hill WL, Ballard S, Coyer MJ, Rowley T (2005) The interaction of testosterone and breeding phase on the reproductive behavior and use of space of male zebra finches. Hormones and Behavior 47: 452-458.

231. Hinde CA (2006) Negotiation over offspring care? a positive response to partner-provisioning rate in great tits. Behavioral Ecology 17: 6-12.

232. Hironaka M, Nomakuchi S, Iwakuma S, Filippi L (2005) Trophic egg production in a subsocial shield bug, Parastrachia japonensis scott (Heteroptera : Parastrachiidae), and its functional value. Ethology 111: 1089-1102.

233. Hirschenhauser K, Oliveira RF (2006) Social modulation of androgens in male vertebrates: meta-analyses of the challenge hypothesis. Animal Behaviour 71: 265-277.

234. Hirschenhauser K, Taborsky M, Oliveira T, Canario AVM, Oliveira RF (2004) A test of the 'challenge hypothesis' in cichlid fish: simulated partner and territory intruder experiments. Animal Behaviour 68: 741-750.

235. Hjernquist MB, Hjernquist KAT, Forsman JT, Gustafsson L (2009) Sex allocation in response to local resource competition over breeding territories. Behavioral Ecology 20: 335-339.

236. Hodge SJ, Flower TP, Clutton-Brock TH (2007) Offspring competition and helper associations in cooperative meerkats. Animal Behaviour 74: 957-964.

237. Hofer H, East ML (2008) Siblicide in Serengeti spotted hyenas: a long-term study of maternal input and cub survival. Behavioral Ecology and Sociobiology 62: 341-351.

238. Hoi-Leitner M, Romero-Pujante M, Hoi H, Pavlova A (2001) Food availability and immune capacity in serin (Serinus serinus) nestlings. Behavioral Ecology and Sociobiology 49: 333-339.

239. Hollander FA, Van Overveld T, Tokka I, Matthysen E (2008) Personality and nest defence in the great tit (Parus major). Ethology 114: 405-412.

240. Horak P (2003) When to pay the cost of reproduction? A brood size manipulation experiment in great tits (Parus major). Behavioral Ecology and Sociobiology 54: 105-112.

241. Horton BM, Holberton RL (2009) Corticosterone manipulations alter morph-specific nestling provisioning behavior in male white-throated sparrows, Zonotrichia albicollis. Hormones and Behavior 56: 510-518.

242. Huang WS (2006) Parental care in the long-tailed skink, Mabuya longicaudata, on a tropical Asian island. Animal Behaviour 72: 791-795.

243. Huang WS (2008) Predation risk of whole-clutch filial cannibalism in a tropical skink with maternal care. Behavioral Ecology 19: 1069-1074.

244. Huang WS, Wang HY (2009) Predation Risks and Anti-Predation Parental Care Behavior: An Experimental Study in a Tropical Skink. Ethology 115: 273-279.

245. Huber S, Millesi E, Dittami JP (2002) Paternal effort and its relation to mating success in the European ground squirrel. Animal Behaviour 63: 157-164.

246. Hume JM, Wynne-Edwards KE (2005) Castration reduces male testosterone, estradiol, and territorial aggression, but not paternal behavior in biparental dwarf hamsters (Phodopus campbelli). Hormones and Behavior 48: 303-310.

247. Hume JM, Wynne-Edwards KE (2006) Paternal responsiveness in biparental dwarf hamsters (Phodopus campbelli) does not require estradiol. Hormones and Behavior 49: 538-544.

248. Humle T, Snowdon CT (2008) Socially biased learning in the acquisition of a complex foraging task in juvenile cottontop tamarins, Saguinus oedipus. Animal Behaviour 75: 267-277.

249. Hunt J, Simmons LW (2002) Behavioural dynamics of biparental care in the dung beetle Onthophagus taurus. Animal Behaviour 64: 65-75.

250. Hunt J, Simmons LW (2004) Optimal maternal investment in the dung beetle Onthophagus taurus? Behavioral Ecology and Sociobiology 55: 302-312.

251. Insley SJ (2001) Mother-Offspring vocal recognition in northern fur seals is mutual but asymmetrical. Animal Behaviour 61: 129-137.

252. Isaksson C, Uller T, Andersson S (2006) Parental effects on carotenoid-based plumage coloration in nestling great tits, Parus major. Behavioral Ecology and Sociobiology 60: 556-562.

253. Itzkowitz M, Santangelo N, Cleveland A, Bockelman A, Richter M (2005) Is the selection of sex-typical parental roles based on an assessment process? A test in the monogamous convict cichlid fish. Animal Behaviour 69: 95-105.

254. Itzkowitz M, Santangelo N, Richter M (2001) Parental division of labour and the shift from minimal to maximal role specializations: an examination using a biparental fish. Animal Behaviour 61: 1237-1245.

255. Itzkowitz M, Santangelo N, Richter M (2002) How similar is the coordination of parental roles among different pairs? An examination of a monogamous fish. Ethology 108: 727-738.

256. Itzkowitz M, Santangelo N, Richter M (2003) How does a parent respond when its mate emphasizes the wrong role? A test using a monogamous fish. Animal Behaviour 66: 863-869.

257. Jacot A, Reers H, Forstmeier W (2010) Individual recognition and potential recognition errors in parent-offspring communication. Behavioral Ecology and Sociobiology 64: 1515-1525.

258. Jacot A, Valcu M, van Oers K, Kempenaers B (2009) Experimental nest site limitation affects reproductive strategies and parental investment in a hole-nesting passerine. Animal Behaviour 77: 1075-1083.

259. Jawor JM, Breitwisch R (2006) Is mate provisioning predicted by ornamentation? A test with northern cardinals (Cardinalis cardinalis). Ethology 112: 888-895.

260. Jawor JM, Gray N, Beall SM, Breitwisch R (2004) Multiple ornaments correlate with aspects of condition and behaviour in female northern cardinals, Cardinalis cardinalis. Animal Behaviour 67: 875-882.

261. Jean-Baptiste N, Terleph TA, Bamshad M (2008) Changes in Paternal Responsiveness of Prairie Voles (Microtus ochrogaster) in Response to Olfactory Cues and Continuous Physical Contact with a Female. Ethology 114: 1239-1246.

262. Jennions MD, Polakow DA (2001) The effect of partial brood loss on male desertion in a cichlid fish: an experimental test. Behavioral Ecology 12: 84-92.

263. Jeon J (2008) Evolution of parental favoritism among different-aged offspring. Behavioral Ecology 19: 344-352.

264. Johnsen A, Delhey K, Schlicht E, Peters A, Kempenaers B (2005) Male sexual attractiveness and parental effort in blue tits: a test of the differential allocation hypothesis. Animal Behaviour 70: 877-888.

265. Johnson JC, Sih A (2007) Fear, food, sex and parental care: a syndrome of boldness in the fishing spider, Dolomedes triton. Animal Behaviour 74: 1131-1138.

266. Johnson LS, Rauch RL, Dellone SN (2004) The process and causes of fledging in a cavity-nesting passerine bird, the house wren (Troglodytes aedon). Ethology 110: 693-705.

267. Johnson M, Aref S, Walters JR (2008) Parent-offspring communication in the western sandpiper. Behavioral Ecology 19: 489-501.

268. Johnstone RA, Hinde CA (2006) Negotiation over offspring care - how should parents respond to each other's efforts? Behavioral Ecology 17: 818-827.

269. Johnstone RA, Roulin A (2003) Sibling negotiation. Behavioral Ecology 14: 780-786.

270. Jones JS, Wynne-Edwards KE (2001) Paternal behaviour in biparental hamsters, Phodopus campbelli, does not require contact with the pregnant female. Animal Behaviour 62: 453-463.

271. Jones KM, Ruxton GD, Monaghan P (2002) Model parents: is full compensation for reduced partner nest attendance compatible with stable biparental care? Behavioral Ecology 13: 838-843.

272. Jones TC, Parker PG (2002) Delayed juvenile dispersal benefits both mother and offspring in the cooperative spider Anelosimus studiosus (Araneae : Theridiidae). Behavioral Ecology 13: 142-148.

273. Jones TC, Riechert SE (2008) Patterns of reproductive success associated with social structure and microclimate in a spider system. Animal Behaviour 76: 2011-2019.

274. Jones TC, Riechert SE, Dalrymple SE, Parker PG (2007) Fostering model explains variation in levels of sociality in a spider system. Animal Behaviour 73: 195-204.

275. Jouventin P, Aubin T (2002) Acoustic systems are adapted to breeding ecologies: individual recognition in nesting penguins. Animal Behaviour 64: 747-757.

276. Juana L, Barbara VG, Martin MT, Agustin C, Guillermo RB, et al. (2010) Neither testosterone levels nor aggression decrease when the male Mongolian gerbil (Meriones unguiculatus) displays paternal behavior. Hormones and Behavior 57: 271-275.

277. Kaitala A, Hardling R, Katvala M, Ordonez RM, Miettinen M (2001) Is nonparental egg carrying parental care? Behavioral Ecology 12: 367-368.

278. Kaptein N, Billen J, Gobin B (2005) Larval begging for food enhances reproductive options in the ponerine ant Gnamptogenys striatula. Animal Behaviour 69: 293-299.

279. Katvala M, Kaitala A (2003) Conspecifics enhance egg production in an egg-carrying bug. Behavioral Ecology 14: 897-901.

280. Kazama K, Watanuki Y (2010) Individual differences in nest defense in the colonial breeding Black-tailed Gulls. Behavioral Ecology and Sociobiology 64: 1239-1246.

281. Keller M, Pawluski JL, Brock O, Douhard Q, Bakker J (2010) The alpha-fetoprotein knock-out mouse model suggests that parental behavior is sexually differentiated under the influence of prenatal estradiol. Hormones and Behavior 57: 434-440.

282. Khan MZ, McNabb FMA, Walters JR, Sharp PJ (2001) Patterns of testosterone and prolactin concentrations and reproductive behavior of helpers and breeders in the cooperatively breeding red-cockaded woodpecker (Picoides borealis). Hormones and Behavior 40: 1-13.

283. Kilner RM (2002) Sex differences in canary (Serinus canaria) provisioning rules. Behavioral Ecology and Sociobiology 52: 400-407.

284. Kilner RM (2003) How selfish is a cowbird nestling? Animal Behaviour 66: 569-576.

285. Kilpi M, Ost M, Lindstrom K, Rita H (2001) Female characteristics and parental care mode in the creching system of eiders, Somateria mollissima. Animal Behaviour 62: 527-534.

286. Kim M, Furness RW, Nager RG (2010) Hatching asynchrony is constrained by parental nest attendance during laying. Behavioral Ecology and Sociobiology 64: 1087-1097.

287. Kim SY, Monaghan P (2005) Interacting effects of nest shelter and breeder quality on behaviour and breeding performance of herring gulls. Animal Behaviour 69: 301-306.

288. Kinahan AA, Pillay N (2008) Dominance status influences female reproductive strategy in a territorial African rodent Rhabdomys pumilio. Behavioral Ecology and Sociobiology 62: 579-587.

289. Kishi S, Nishida T (2006) Adjustment of parental investment in the dung beetle Onthophagus atripennis (Col., Scarabaeidae). Ethology 112: 1239-1245.

290. Kishi S, Nishida T (2008) Optimal investment in sons and daughters when parents do not know the sex of their offspring. Behavioral Ecology and Sociobiology 62: 607-615.

291. Kitaysky AS, Romano MD, Piatt JF, Wingfield JC, Kikuchi M (2005) The adrenocortical response of tufted puffin chicks to nutritional deficits. Hormones and Behavior 47: 606-619.

292. Kitaysky AS, Wingfield JC, Piatt JF (2001) Corticosterone facilitates begging and affects resource allocation in the black-legged kittiwake. Behavioral Ecology 12: 619-625.

293. Kleindorfer S, Fessl B, Hoi H (2005) Avian nest defence behaviour: assessment in relation to predator distance and type, and nest height. Animal Behaviour 69: 307-313.

294. Klug H, Chin A, Mary C (2005) The net effects of guarding on egg survivorship in the flagfish, Jordanella floridaei. Animal Behaviour 69: 661-668.

295. Knornschild M, Von Helversen O (2008) Nonmutual vocal mother-pup recognition in the greater sac-winged bat. Animal Behaviour 76: 1001-1009.

296. Kober M, Trillmich F, Naguib M (2007) Vocal mother-pup communication in guinea pigs: effects of call familiarity and female reproductive state. Animal Behaviour 73: 917-925.

297. Koch KA, Wingfield JC, Buntin JD (2002) Glucocorticoids and parental hyperphagia in ring doves (Streptopelia risoria). Hormones and Behavior 41: 9-21.

298. Koch KA, Wingfield JC, Buntin JD (2004) Prolactin-induced parental hyperphagia in ring doves: are glucocorticoids involved? Hormones and Behavior 46: 498-505.

299. Koenig WD, Stanback MT, Haydock J, Kraaijeveld-Smit F (2001) Nestling sex ratio variation in the cooperatively breeding acorn woodpecker (Melanerpes formicivorus). Behavioral Ecology and Sociobiology 49: 357-365.

300. Kokko H, Johnstone RA, Wright J (2002) The evolution of parental and alloparental effort in cooperatively breeding groups: when should helpers pay to stay? Behavioral Ecology 13: 291-300.

301. Kolliker M (2007) Benefits and costs of earwig (Forficula auricularia) family life. Behavioral Ecology and Sociobiology 61: 1489-1497.

302. Kolliker M, Richner H (2001) Parent-offspring conflict and the genetics of offspring solicitation and parental response. Animal Behaviour 62: 395-407.

303. Kolliker M, Richner H (2004) Navigation in a cup: chick positioning in great tit, Parus major, nests. Animal Behaviour 68: 941-948.

304. Kondoh M, Okuda N (2002) Mate availability influences filial cannibalism in fish with paternal care. Animal Behaviour 63: 227-233.

305. Kontiainen P, Pietiainen H, Huttunen K, Karell P, Kolunen H, et al. (2009) Aggressive Ural owl mothers recruit more offspring. Behavioral Ecology 20: 789-796.

306. Kopisch AD, Schwagmeyer PL, Mock DW (2005) Individual consistency in parental effort across multiple stages of care in the house sparrow, Passer domesticus. Ethology 111: 1062-1070.

307. Korb J, Aanen DK (2003) The evolution of uniparental transmission of fungal symbionts in fungus-growing termites (Macrotermitinae). Behavioral Ecology and Sociobiology 53: 65-71.

308. Kosztolanyi A, Cuthill IC, Szekely T (2009) Negotiation between parents over care: reversible compensation during incubation. Behavioral Ecology 20: 446-452.

309. Kosztolanyi A, Szekely T, Cuthill IC (2003) Why do both parents incubate in the Kentish plover? Ethology 109: 645-658.

310. Kral TVE, Rauh EM (2010) Eating behaviors of children in the context of their family environment. Physiology & Behavior 100: 567-573.

311. Krams I, Berzins A, Krama T (2009) Group effect in nest defence behaviour of breeding pied flycatchers, Ficedula hypoleuca. Animal Behaviour 77: 513-517.

312. Krebs EA (2001) Begging and food distribution in crimson rosella (Platycercus elegans) broods: why don't hungry chicks beg more? Behavioral Ecology and Sociobiology 50: 20-30.

313. Krebs EA, Putland DA (2004) Chic chicks: the evolution of chick ornamentation in rails. Behavioral Ecology 15: 946-951.

314. Krist M, Grim T (2007) Are blue eggs a sexually selected signal of female collared flycatchers? A cross-fostering experiment. Behavioral Ecology and Sociobiology 61: 863-876.

315. Kudo S (2002) Phenotypic selection and function of reproductive behavior in the subsocial bug Elasmucha putoni (Heteroptera : Acanthosomatidae). Behavioral Ecology 13: 742-749.

316. Kurata A, Morinobu S, Fuchikami M, Yamamoto S, Yamawaki S (2009) Maternal postpartum learned helplessness (LH) affects maternal care by dams and responses to the LH test in adolescent offspring. Hormones and Behavior 56: 112-120.

317. Kuzawa CW, Gettler LT, Huang YY, McDade TW (2010) Mothers have lower testosterone than non-mothers: Evidence from the Philippines. Hormones and Behavior 57: 441-447.

318. Kuzawa CW, Gettler LT, Muller MN, McDade TW, Feranil AB (2009) Fatherhood, pairbonding and testosterone in the Philippines. Hormones and Behavior 56: 429-435.

319. Kvarnemo C (2006) Evolution and maintenance of male care: is increased paternity a neglected benefit of care? Behavioral Ecology 17: 144-148.

320. Landete-Castillejos T, Garcia A, Lopez-Serrano FR, Gallego L (2005) Maternal quality and differences in milk production and composition for male and female Iberian red deer calves (Cervus elaphus hispanicus). Behavioral Ecology and Sociobiology 57: 267-274.

321. Langmore NE, Cockburn A, Russell AF, Kilner RM (2009) Flexible cuckoo chick-rejection rules in the superb fairy-wren. Behavioral Ecology 20: 978-984.

322. Lappan S (2008) Male care of infants in a siamang (Symphalangus syndactylus) population including socially monogamous and polyandrous groups. Behavioral Ecology and Sociobiology 62: 1307-1317.

323. Laurien-Kehnen C, Trillmich F (2003) Lactation performance of guinea pigs (Cavia porcellus) does not respond to experimental manipulation of pup demands. Behavioral Ecology and Sociobiology 53: 145-152.

324. Lawes MJ, Marthews TR (2003) When will rejection of parasite nestlings by hosts of nonevicting avian brood parasites be favored? A misimprinting-equilibrium model. Behavioral Ecology 14: 757-770.

325. Le Bohec C, Gauthier-Clerc M, Le Maho Y (2005) The adaptive significance of creches in the king penguin. Animal Behaviour 70: 527-538.

326. Lee AW, Brown RE (2007) Comparison of medial preoptic, amygdala, and nucleus accumbens lesions on parental behavior in California mice (Peromyscus californicus). Physiology & Behavior 92: 617-628.

327. Leech DI, Rowe LV, Hartley IR (2006) Experimental evidence for adjustment of parental investment in relation to brood sex ratio in the blue tit. Animal Behaviour 72: 1301-1307.

328. Leedman AW, Magrath RD (2003) Long-term brood division and exclusive parental care in a cooperatively breeding passerine. Animal Behaviour 65: 1093-1108.

329. Lehtonen TK, Lindstrom K (2007) Mate compatibility, parental allocation and fitness consequences of mate choice in the sand goby Pomatoschistus minutus. Behavioral Ecology and Sociobiology 61: 1581-1588.

330. Leitner S, Marshall RC, Leisler B, Catchpole CK (2006) Male song quality, egg size and offspring sex in captive canaries (Serinus canaria). Ethology 112: 554-563.

331. Lendvai AZ, Barta Z, Chastel O (2009) Conflict over parental care in house sparrows: do females use a negotiation rule? Behavioral Ecology 20: 651-656.

332. Lendvai AZ, Chastel O (2008) Experimental mate-removal increases the stress response of female house sparrows: The effects of offspring value? Hormones and Behavior 53: 395-401.

333. Lendvai AZ, Chastel O (2010) Natural variation in stress response is related to post-stress parental effort in male house sparrows. Hormones and Behavior 58: 936-942.

334. Lengyel S (2007) Benefits of large broods by higher chick survival and better territories in a precocial shorebird. Behavioral Ecology and Sociobiology 61: 589-598.

335. Leonard ML, Horn AG (2001) Acoustic signalling of hunger and thermal state by nestling tree swallows. Animal Behaviour 61: 87-93.

336. Leonard ML, Horn AG (2001) Begging calls and parental feeding decisions in tree swallows (Tachycineta bicolor). Behavioral Ecology and Sociobiology 49: 170-175.

337. Leonard ML, Horn AG (2001) Dynamics of calling by tree swallow (Tachycineta bicolor) nestmates. Behavioral Ecology and Sociobiology 50: 430-435.

338. Leonard ML, Horn AG (2006) Age-related changes in signalling of need by nestling tree swallows (Tachycineta bicolor). Ethology 112: 1020-1026.

339. Leonard ML, Horn AG (2008) Does ambient noise affect growth and begging call structure in nestling birds? Behavioral Ecology 19: 502-507.

340. Leonard ML, Horn AG, Mukhida A (2005) False alarms and begging in nestling birds. Animal Behaviour 69: 701-708.

341. Leonard ML, Horn AG, Parks E (2003) The role of posturing and calling in the begging display of nestling birds. Behavioral Ecology and Sociobiology 54: 188-193.

342. Leonard ML, Horn AG, Porter J (2003) Does begging affect growth in nestling tree swallows, Tachycineta bicolor? Behavioral Ecology and Sociobiology 54: 573-577.

343. Lessells CM, Poelman EH, Mateman AC, Cassey P (2006) Consistent feeding positions of great tit parents. Animal Behaviour 72: 1249-1257.

344. Lewis S, Hamer KC, Money L, Griffiths R, Wanless S, et al. (2004) Brood neglect and contingent foraging behavior in a pelagic seabird. Behavioral Ecology and Sociobiology 56: 81-88.

345. Li DQ, Jackson RR (2003) A predator's preference for egg-carrying prey: a novel cost of parental care. Behavioral Ecology and Sociobiology 55: 129-136.

346. Li QL, Zhang L (2010) Parent-offspring recognition in Brandt's voles, Lasiopodomys brandti. Animal Behaviour 79: 797-801.

347. Li SH, Brown JL (2002) Reduction of maternal care: a new benefit of multiple mating? Behavioral Ecology 13: 87-93.

348. Lichtenstein G (2001) Low success of shiny cowbird chicks parasitizing rufous-bellied thrushes: chick-chick competition or parental discrimination? Animal Behaviour 61: 401-413.

349. Lichtenstein G (2001) Selfish begging by screaming cowbirds, a mimetic brood parasite of the bay-winged cowbird. Animal Behaviour 61: 1151-1158.

350. Lichtenstein G, Dearborn DC (2004) Begging and short-term need in cowbird nestlings: how different are brood parasites? Behavioral Ecology and Sociobiology 56: 352-359.

351. Ligon RA, Hill GE (2010) Feeding decisions of eastern bluebirds are situationally influenced by fledgling plumage color. Behavioral Ecology 21: 456-464.

352. Ligon RA, Hill GE (2010) Sex-biased parental investment is correlated with mate ornamentation in eastern bluebirds. Animal Behaviour 79: 727-734.

353. Limmer B, Becker PH (2009) Improvement in chick provisioning with parental experience in a seabird. Animal Behaviour 77: 1095-1101.

354. Lindstrom K, St Mary C, Pampoulie C (2006) Sexual selection for male parental care in the sand goby, Pomatoschistus minutus. Behavioral Ecology and Sociobiology 60: 46-51.

355. Lislevand T, Byrkjedal I, Gronstol GB, Hafsmo JE, Kallestad GR, et al. (2004) Incubation behaviour in Northern Lapwings: Nocturnal nest attentiveness and possible importance of individual breeding quality. Ethology 110: 177-192.

356. Lissaker M, Kvarnemo C (2006) Ventilation or nest defense - parental care trade-offs in a fish with male care. Behavioral Ecology and Sociobiology 60: 864-873.

357. Lissaker M, Kvarnemo C, Svensson O (2003) Effects of a low oxygen environment on parental effort and filial cannibalism in the male sand goby, Pomatoschistus minutus. Behavioral Ecology 14: 374-381.

358. Lissaker M, Svensson O (2008) Cannibalize or care? The role of perceived paternity in the sand goby, Pomatoschistus minutus. Behavioral Ecology and Sociobiology 62: 1467-1475.

359. Lloyd JD, Martin TE (2004) Nest-site preference and maternal effects on offspring growth. Behavioral Ecology 15: 816-823.

360. Loiseau C, Fellous S, Haussy C, Chastel O, Sorci G (2008) Condition-dependent effects of corticosterone on a carotenoid-based begging signal in house sparrows. Hormones and Behavior 53: 266-273.

361. Londono GA, Levey DJ, Robinson SK (2008) Effects of temperature and food on incubation behaviour of the northern mockingbird, Mimus polyglottos. Animal Behaviour 76: 669-677.

362. Lonstein JS, Rood BD, De Vries GJ (2002) Parental responsiveness is feminized after neonatal castration in virgin male prairie voles, but is not masculinized by perinatal testosterone in virgin females. Hormones and Behavior 41: 80-87.

363. Lopez-Rull I, Celis P, Gil D (2007) Egg colour covaries with female expression of a male ornament in the spotless starling (Sturnus unicolor). Ethology 113: 926-933.

364. Losdat S, Helfenstein F, Gaude B, Richner H (2010) Effect of sibling competition and male carotenoid supply on offspring condition and oxidative stress. Behavioral Ecology 21: 1271-1277.

365. Low M, Joy MK, Makan T (2006) Using regression trees to predict patterns of male provisioning in the stitchbird (hihi). Animal Behaviour 71: 1057-1068.

366. Lynn SE, Hayward LS, Benowitz-Fredericks ZM, Wingfield JC (2002) Behavioural insensitivity to supplementary testosterone during the parental phase in the chestnut-collared longspur, - Calcarius ornatus. Animal Behaviour 63: 795-803.

367. Lynn SE, Walker BG, Wingfield JC (2005) A phylogenetically controlled test of hypotheses for behavioral insensitivity to testosterone in birds. Hormones and Behavior 47: 170-177.

368. MacColl ADC, Hatchwell BJ (2003) Sharing of caring: nestling provisioning behaviour of long-tailed tit, Aegithalos caudatus, parents and helpers. Animal Behaviour 66: 955-964.

369. Macedo RHF, Cariello MO, Pacheco AM, Schwabl HG (2004) Significance of social parameters on differential nutrient investment in guira cuckoo, Guira guira, eggs. Animal Behaviour 68: 685-694.

370. MacFarlane GR, Blomberg SP, Vasey PL (2010) Homosexual behaviour in birds: frequency of expression is related to parental care disparity between the sexes. Animal Behaviour 80: 375-390.

371. MacGregor NA, Cockburn A (2002) Sex differences in parental response to begging nestlings in superb fairy-wrens. Animal Behaviour 63: 923-932.

372. Madden JR, Kilner RM, Davies NB (2005) Nestling responses to adult food and alarm calls: 1. Species-specific responses in two cowbird hosts. Animal Behaviour 70: 619-627.

373. Madden JR, Kilner RM, Davies NB (2005) Nestling responses to adult food and alarm calls: 2. Cowbirds and red-winged blackbirds reared by eastern phoebe hosts. Animal Behaviour 70: 629-637.

374. Madden JR, Kunc HJP, English S, Clutton-Brock TH (2009) Why do meerkat pups stop begging? Animal Behaviour 78: 85-89.

375. Madden JR, Kunc HJP, English S, Manser MB, Clutton-Brock TH (2009) Do meerkat (Suricata suricatta) pups exhibit strategic begging behaviour and so exploit adults that feed at relatively high rates? Behavioral Ecology and Sociobiology 63: 1259-1268.

376. Maestripieri D (2001) Intraspecific variability in parenting styles of rhesus macaques (Macaca mulatta): The role of the social environment. Ethology 107: 237-248.

377. Magee SE, Neff BD (2006) Temporal variation in decisions about parental care in bluegill, Lepomis macrochirus. Ethology 112: 1000-1007.

378. Magee SE, Neff BD, Knapp R (2006) Plasma levels of androgens and cortisol in relation to breeding behavior in parental male bluegill sunfish, Lepomis macrochirus. Hormones and Behavior 49: 598-609.

379. Magrath MJL, Brouwer L, Komdeur J (2003) Egg size and laying order in relation to offspring sex in the extreme sexually size dimorphic brown songlark, Cinclorhamphus cruralis. Behavioral Ecology and Sociobiology 54: 240-248.

380. Magrath RD, Pitcher BJ, Dalziell AH (2007) How to be fed but not eaten: nestling responses to parental food calls and the sound of a predator's footsteps. Animal Behaviour 74: 1117-1129.

381. Malueg AL, Walters JR, Moore IT (2009) Do stress hormones suppress helper reproduction in the cooperatively breeding red-cockaded woodpecker (Picoides borealis)? Behavioral Ecology and Sociobiology 63: 687-698.

382. Manica A (2002) Alternative strategies for a father with a small brood: mate, cannibalise or care. Behavioral Ecology and Sociobiology 51: 319-323.

383. Manica A (2004) Parental fish change their cannibalistic behaviour in response to the cost-to-benefit ratio of parental care. Animal Behaviour 67: 1015-1021.

384. Manser MB, Madden JR, Kunc HP, English S, Clutton-Brock T (2008) Signals of need in a cooperatively breeding mammal with mobile offspring. Animal Behaviour 76: 1805-1813.

385. Margulis SW, Nabong M, Alaks G, Walsh A, Lacy RC (2005) Effects of early experience on subsequent parental behaviour and reproductive success in oldfield mice, Peromyscus polionotus. Animal Behaviour 69: 627-634.

386. Martin-Galvez D, Soler M, Soler JJ, Martin-Vivaldi M, Palomino JJ (2005) Food acquisition by common cuckoo chicks in rufous bush robin nests and the advantage of eviction behaviour. Animal Behaviour 70: 1313-1321.

387. Martinez-Padilla J (2006) Prelaying maternal condition modifies the association between egg mass and T cell-mediated immunity in kestrels. Behavioral Ecology and Sociobiology 60: 510-515.

388. Martinez-Padilla J, Fargallo JA (2007) Food supply during prelaying period modifies the sex-dependent investment in eggs of Eurasian kestrels. Behavioral Ecology and Sociobiology 61: 1735-1742.

389. Mas F, Kolliker M (2008) Maternal care and offspring begging in social insects: chemical signalling, hormonal regulation and evolution. Animal Behaviour 76: 1121-1131.

390. Matsumoto K, Yanagisawa Y (2001) Monogamy and sex role reversal in the pipefish Corythoichthys haematopterus. Animal Behaviour 61: 163-170.

391. Matysiokova B, Remes V (2010) Incubation Feeding and Nest Attentiveness in a Socially Monogamous Songbird: Role of Feather Colouration, Territory Quality and Ambient Environment. Ethology 116: 596-607.

392. Maurer G (2008) Who cares? Males provide most parental care in a monogamous nesting cuckoo. Ethology 114: 540-547.

393. Maurer G, Magrath RD, Leonard ML, Horn AG, Donnelly C (2003) Begging to differ: scrubwren nestlings beg to alarm calls and vocalize when parents are absent. Animal Behaviour 65: 1045-1055.

394. McDonald PG, Buttemer WA, Astheimer LB (2001) The influence of testosterone on territorial defence and parental behavior in male free-living rufous whistlers, Pachycephala rufiventris. Hormones and Behavior 39: 185-194.

395. McDonald PG, Kazem AJN, Wright J (2009) Cooperative provisioning dynamics: fathers and unrelated helpers show similar responses to manipulations of begging. Animal Behaviour 77: 369-376.

396. McDonald PG, Olsen PD, Cockburn A (2005) Sex allocation and nestling survival in a dimorphic raptor: does size matter? Behavioral Ecology 16: 922-930.

397. McDonald PG, Wilson DR, Evans CS (2009) Nestling begging increases predation risk, regardless of spectral characteristics or avian mobbing. Behavioral Ecology 20: 821-829.

398. McDonald-Madden E, Elgar MA, Handasyde KA (2004) Responses to threat by female bobucks, Trichosurus caninus, during different stages of offspring development. Behavioral Ecology and Sociobiology 56: 322-327.

399. McNamara JM, Houston AI, Barta Z, Osorno JL (2003) Should young ever be better off with one parent than with two? Behavioral Ecology 14: 301-310.

400. McNamara JM, Houston AI, Szekely T, Webb JN (2002) Do parents make independent decisions about desertion? Animal Behaviour 64: 147-149.

401. Meddle SL, Romero LM, Astheimer LB, Buttemer WA, Moore IT, et al. (2002) Steroid hormone interrelationships with territorial aggression in an arctic-breeding songbird, Gambel's white-crowned sparrow, Zonotrichia leucophrys gambelii. Hormones and Behavior 42: 212-221.

402. Mennerat A, Perret P, Bourgault P, Blondel J, Gimenez O, et al. (2009) Aromatic plants in nests of blue tits: positive effects on nestlings. Animal Behaviour 77: 569-574.

403. Miller DA, Vleck CM, Otis DL (2009) Individual variation in baseline and stress-induced corticosterone and prolactin levels predicts parental effort by nesting mourning doves. Hormones and Behavior 56: 457-464.

404. Mitchell DP, Dunn PO, Whittingham LA, Freeman-Gallant CR (2007) Attractive males provide less parental care in two populations of the common yellowthroat. Animal Behaviour 73: 165-170.

405. Mock DW, Schwagmeyer PL, Dugas MB (2009) Parental provisioning and nestling mortality in house sparrows. Animal Behaviour 78: 677-684.

406. Mock DW, Schwagmeyer PL, Parker GA (2005) Male house sparrows deliver more food to experimentally subsidized offspring. Animal Behaviour 70: 225-236.

407. Moller AP (2006) Rapid change in nest size of a bird related to change in a secondary sexual character. Behavioral Ecology 17: 108-116.

408. Moody AT, Wilhelm SI, Cameron-MacMillan ML, Walsh CJ, Storey AE (2005) Divorce in common murres (Uria aalge): relationship to parental quality. Behavioral Ecology and Sociobiology 57: 224-230.

409. Moreno J, Lobato E, Merino S, Martinez-de La Puente J (2008) Blue-Green Eggs in Pied Flycatchers: An Experimental Demonstration that a Supernormal Stimulus Elicits Improved Nestling Condition. Ethology 114: 1078-1083.

410. Moreno J, Veiga JP, Romasanta M, Sanchez S (2002) Effects of maternal quality and mating status on female reproductive success in the polygynous spotless starling. Animal Behaviour 64: 197-206.

411. Moreno-Rueda G (2007) Yolk androgen deposition as a female tactic to manipulate paternal contribution. Behavioral Ecology 18: 496-498.

412. Morton ES, Stutchbury BJM, Chiver I (2010) Parental conflict and brood desertion by females in blue-headed vireos. Behavioral Ecology and Sociobiology 64: 947-954.

413. Moskat C, Hauber ME, Aviles JM, Ban M, Hargitai R, et al. (2009) Increased host tolerance of multiple cuckoo eggs leads to higher fledging success of the brood parasite. Animal Behaviour 77: 1281-1290.

414. Mota MTD, Franci CR, de Sousa MBC (2006) Hormonal changes related to paternal and alloparental care in common marmosets (Callithrix jacchus). Hormones and Behavior 49: 293-302.

415. Muck C, Kempenaers B, Kuhn S, Valcu M, Goymann W (2009) Paternity in the classical polyandrous black coucal (Centropus grillii)-a cuckoo accepting cuckoldry? Behavioral Ecology 20: 1185-1193.

416. Mulard H, Aubin T, White JF, Hatch SA, Danchin E (2008) Experimental evidence of vocal recognition in young and adult black-legged kittiwakes. Animal Behaviour 76: 1855-1861.

417. Mulard H, Vignal C, Pelletier L, Blanc A, Mathevon N (2010) From preferential response to parental calls to sex-specific response to conspecific calls in juvenile zebra finches. Animal Behaviour 80: 189-195.

418. Muller CA, Manser MB (2008) Mutual recognition of pups and providers in the cooperatively breeding banded mongoose. Animal Behaviour 75: 1683-1692.

419. Muller JK, Eggert AK, Elsner T (2003) Nestmate recognition in burying beetles: the "breeder's badge" as a cue used by females to distinguish their mates from male intruders. Behavioral Ecology 14: 212-220.

420. Muller W, Boonen S, Groothuis TGG, Eens M (2010) Maternal yolk testosterone in canary eggs: toward a better understanding of mechanisms and function. Behavioral Ecology 21: 493-500.

421. Muller W, Deptuch K, Lopez-Rull I, Gil D (2007) Elevated yolk androgen levels benefit offspring development in a between-clutch context. Behavioral Ecology 18: 929-936.

422. Munguia-Steyer R, Favila ME, Macias-Ordonez R (2008) Brood pumping modulation and the benefits of paternal care in Abedus breviceps (Hemiptera : Belostomatidae). Ethology 114: 693-700.

423. Murphy TG (2007) Dishonest 'preemptive' pursuit-deterrent signal? Why the turquoise-browed motmot wags its tail before feeding nestlings. Animal Behaviour 73: 965-970.

424. Murray CM, Lonsdorf EV, Eberly LE, Pusey AE (2009) Reproductive energetics in free-living female chimpanzees (Pan troglodytes schweinfurthii). Behavioral Ecology 20: 1211-1216.

425. Nakagawa S, Ockendon N, Gillespie DOS, Hatchwell BJ, Burke T (2007) Assessing the function of house sparrows' bib size using a flexible meta-analysis method. Behavioral Ecology 18: 831-840.

426. Nakagawa S, Waas JR, Miyazaki M (2001) Heart rate changes reveal that little blue penguin chicks (Eudyptula minor) can use vocal signatures to discriminate familiar from unfamiliar chicks. Behavioral Ecology and Sociobiology 50: 180-188.

427. Nathan A, Legge S, Cockburn A (2001) Nestling aggression in broods of a siblicidal kingfisher, the laughing kookaburra. Behavioral Ecology 12: 716-725.

428. Nazareth TM, Machado G (2010) Mating system and exclusive postzygotic paternal care in a Neotropical harvestman (Arachnida: Opiliones). Animal Behaviour 79: 547-554.

429. Neff BD (2003) Paternity and condition affect cannibalistic behavior in nest-tending bluegill sunfish. Behavioral Ecology and Sociobiology 54: 377-384.

430. Neff BD, Cargnelli LM, Cote IM (2004) Solitary nesting as an alternative breeding tactic in colonial nesting bluegill sunfish (Lepomis macrochirus). Behavioral Ecology and Sociobiology 56: 381-387.

431. Neff BD, Knapp R (2009) Paternity, parental behavior and circulating steroid hormone concentrations in nest-tending male bluegill. Hormones and Behavior 56: 239-245.

432. Neff BD, Sherman PW (2005) In vitro fertilization reveals offspring recognition via self-referencing in a fish with paternal care and cuckoldry. Ethology 111: 425-438.

433. Nettle D (2010) Dying young and living fast: variation in life history across English neighborhoods. Behavioral Ecology 21: 387-395.

434. Neuenschwander S, Brinkhof MWG, Kolliker M, Richner H (2003) Brood size, sibling competition, and the cost of begging in great tits (Parus major). Behavioral Ecology 14: 457-462.

435. Nguyen N, Gesquiere LR, Wango EO, Alberts SC, Altmann J (2008) Late pregnancy glucocorticoid levels predict responsiveness in wild baboon mothers (Papio cynocephalus). Animal Behaviour 75: 1747-1756.

436. Nguyen N, Van Horn RC, Alberts SC, Altmann J (2009) "Friendships" between new mothers and adult males: adaptive benefits and determinants in wild baboons (Papio cynocephalus). Behavioral Ecology and Sociobiology 63: 1331-1344.

437. Nielsen CR, Parker PG, Gates RJ (2006) Intraspecific nest parasitism of cavity-nesting wood ducks: costs and benefits to hosts and parasites. Animal Behaviour 72: 917-926.

438. Nilsson JA (2003) Ectoparasitism in marsh tits: costs and functional explanations. Behavioral Ecology 14: 175-181.

439. Noguera JC, Morales J, Perez C, Velando A (2010) On the oxidative cost of begging: antioxidants enhance vocalizations in gull chicks. Behavioral Ecology 21: 479-484.

440. Nordstrom M, Laine J, Ahola M, Korpimaki E (2004) Reduced nest defence intensity and improved breeding success in terns as responses to removal of non-native American mink. Behavioral Ecology and Sociobiology 55: 454-460.

441. Nunes S, Fite JE, Patera KJ, French JA (2001) Interactions among paternal behavior, steroid hormones, and parental experience in male marmosets (Callithrix kuhlii). Hormones and Behavior 39: 70-82.

442. O'Connor DE, Shine R (2004) Parental care protects against infanticide in the lizard Egernia saxatilis (Scincidae). Animal Behaviour 68: 1361-1369.

443. O'Neal DM, Reichard DG, Pavilis K, Ketterson ED (2008) Experimentally-elevated testosterone, female parental care, and reproductive success in a songbird, the Dark-eyed Junco (Junco hyemalis). Hormones and Behavior 54: 571-578.

444. Olsen BJ, Greenberg R, Fleischer RC, Walters JR (2008) Extrapair paternity in the swamp sparrow, Melospiza georgiana: male access or female preference? Behavioral Ecology and Sociobiology 63: 285-294.

445. Ortolani I, Zechini L, Turillazzi S, Cervo R (2010) Recognition of a paper wasp social parasite by its host: evidence for a visual signal reducing host aggressiveness. Animal Behaviour 80: 683-688.

446. Osorio-Beristain H, Drummond H (2001) Male boobies expel eggs when paternity is in doubt. Behavioral Ecology 12: 16-21.

447. Osorio-Beristain M, Perez-Staples D, Drummond H (2006) Does booby egg dumping amount to quasi-parasitism? Ethology 112: 625-630.

448. Osorno JL, Szekely T (2004) Sexual conflict and parental care in magnificent frigatebirds: full compensation by deserted females. Animal Behaviour 68: 337-342.

449. Ost M, Back A (2003) Spatial structure and parental aggression in eider broods. Animal Behaviour 66: 1069-1075.

450. Ost M, Jaatinen K, Steele B (2007) Aggressive females seize central positions and show increased vigilance in brood-rearing coalitions of eiders. Animal Behaviour 73: 239-247.

451. Ost M, Mantila L, Kilpi M (2002) Shared care provides time-budgeting advantages for female eiders. Animal Behaviour 64: 223-231.

452. Ost M, Ydenberg R, Kilpi M, Lindstrom K (2003) Condition and coalition formation by brood-rearing common eider females. Behavioral Ecology 14: 311-317.

453. Ost M, Ydenberg R, Lindstrom K, Kilpi M (2003) Body condition and the grouping behavior of brood-caring female common eiders (Somateria mollissima). Behavioral Ecology and Sociobiology 54: 451-457.

454. Ostlund-Nilsson S (2002) Does paternity or paternal investment determine the level of paternal care and does female choice explain egg stealing in the fifteen-spined stickleback? Behavioral Ecology 13: 188-192.

455. Ostreiher R (2001) The importance of nestling location for obtaining food in open cup-nests. Behavioral Ecology and Sociobiology 49: 340-347.

456. Ostreiher R (2003) Is mobbing altruistic or selfish behaviour? Animal Behaviour 66: 145-149.

457. Otali E, Gilchrist JS (2006) Why chimpanzee (Pan troglodytes schweinfurthii) mothers are less gregarious than nonmothers and males: the infant safety hypothesis. Behavioral Ecology and Sociobiology 59: 561-570.

458. Pacheco ML, McDonald PG, Wright J, Kazem AJN, Clarke MF (2008) Helper contributions to antiparasite behavior in the cooperatively breeding bell miner. Behavioral Ecology 19: 558-566.

459. Pall MK, Mayer I, Borg B (2002) Androgen and behavior in the male three-spined stickleback, Gasterosteus aculeatus I. - Changes in 11-ketotestosterone levels during the nesting cycle. Hormones and Behavior 41: 377-383.

460. Pall MK, Mayer I, Borg B (2002) Androgen and Behavior in the male three-spined stickleback, Gasterosteus aculeatus II. Castration and 11-ketoandrostenedione effects on courtship and parental care during the nesting cycle. Hormones and Behavior 42: 337-344.

461. Pampoulie C, Lindstrom K, St Mary CM (2004) Have your cake and eat it too: male sand gobies show more parental care in the presence of female partners. Behavioral Ecology 15: 199-204.

462. Paredes R, Jones IL, Boness DJ (2005) Reduced parental care, compensatory behaviour and reproductive costs of thick-billed murres equipped with data loggers. Animal Behaviour 69: 197-208.

463. Parejo D, Danchin E (2006) Brood size manipulation affects frequency of second clutches in the blue tit. Behavioral Ecology and Sociobiology 60: 184-194.

464. Parejo D, Perez-Contreras T, Navarro C, Soler JJ, Aviles JM (2008) Spotless starlings rely on public information while visiting conspecific nests: an experiment. Animal Behaviour 75: 483-488.

465. Patterson L, Dick JTA, Elwood RW (2008) Embryo retrieval and kin recognition in an amphipod (Crustacea). Animal Behaviour 76: 717-722.

466. Pavel V, Bures S (2001) Offspring age and nest defence: test of the feedback hypothesis in the meadow pipit. Animal Behaviour 61: 297-303.

467. Payne RB, Woods JL, Payne LL (2001) Parental care in estrildid finches: experimental tests of a model of Vidua brood parasitism. Animal Behaviour 62: 473-483.

468. Peluc SI, Sillett TS, Rotenberry JT, Ghalambor CK (2008) Adaptive phenotypic plasticity in an island songbird exposed to a novel predation risk. Behavioral Ecology 19: 830-835.

469. Penteriani V, Delgado MD, Alonso-Alvarez C, Pina NV, Sergio F, et al. (2007) The importance of visual cues for nocturnal species: Eagle owl fledglings signal with white mouth feathers. Ethology 113: 934-943.

470. Perry JC, Roitberg BD (2005) Ladybird mothers mitigate offspring starvation risk by laying trophic eggs. Behavioral Ecology and Sociobiology 58: 578-586.

471. Peters A (2002) Testosterone and the trade-off between mating and paternal effort in extrapair-mating superb fairy-wrens. Animal Behaviour 64: 103-112.

472. Peters A, Cockburn A, Cunningham R (2002) Testosterone treatment suppresses paternal care in superb fairy-wrens, Malurus cyaneus, despite their concurrent investment in courtship. Behavioral Ecology and Sociobiology 51: 538-547.

473. Peterson JH, Roitberg BD (2006) Impacts of flight distance on sex ratio and resource allocation to offspring in the leafcutter bee, Megachile rotundata. Behavioral Ecology and Sociobiology 59: 589-596.

474. Peterson KA, Thusius KJ, Whittingham LA, Dunn PO (2001) Allocation of male parental care in relation to paternity within and among broods of the common yellowthroat (Geothlypis trichas). Ethology 107: 573-586.

475. Pflanz T (2002) Age and brood defence in male CRL : NMRI BR laboratory mice, Mus musculus domesticus. Animal Behaviour 63: 613-616.

476. Pierce EP, Oring LW, Roskaft E, Lifjeld JT (2010) Why don't female purple sandpipers perform brood care? A removal experiment. Behavioral Ecology 21: 275-283.

477. Pike TW, Blount JD, Lindstrom J, Metcalfe NB (2007) Dietary carotenoid availability influences a male's ability to provide parental care. Behavioral Ecology 18: 1100-1105.

478. Pilastro A, Griggio M, Matessi G (2003) Male rock sparrows adjust their breeding strategy according to female ornamentation: parental or mating investment? Animal Behaviour 66: 265-271.

479. Pilz KM, Quiroga M, Schwabl H, Adkins-Regan E (2004) European starling chicks benefit from high yolk testosterone levels during a drought year. Hormones and Behavior 46: 179-192.

480. Pilz KM, Smith HG, Andersson M (2005) Brood parasitic European starlings do not lay high-quality eggs. Behavioral Ecology 16: 507-513.

481. Pilz KM, Smith HG, Sandell MI, Schwabl H (2003) Interfemale variation in egg yolk androgen allocation in the European starling: do high-quality females invest more? Animal Behaviour 65: 841-850.

482. Porkert J, Spinka M (2006) Begging in common redstart nestlings: Scramble competition or signalling of need? Ethology 112: 398-410.

483. Potvin DA, MacDougall-Shackleton EA (2009) Parental investment amplifies effects of genetic complementarity on growth rates in song sparrows, Melospiza melodia. Animal Behaviour 78: 943-948.

484. Poysa H (2004) Relatedness and the evolution of conspecific brood parasitism: parameterizing a model with data for a precocial species. Animal Behaviour 67: 673-679.

485. Poysa H, Lindblom K, Rutila J, Sorjonen J (2010) Response of parasitically laying goldeneyes to experimental nest predation. Animal Behaviour 80: 881-886.

486. Prato-Previde E, Fallani G, Valsecchi P (2006) Gender differences in owners interacting with pet dogs: An observational study. Ethology 112: 64-73.

487. Preault M, Chastel O, Cezilly F, Faivre B (2005) Male bill colour and age are associated with parental abilities and breeding performance in blackbirds. Behavioral Ecology and Sociobiology 58: 497-505.

488. Pryke SR, Griffith SC (2010) Maternal adjustment of parental effort in relation to mate compatibility affects offspring development. Behavioral Ecology 21: 226-232.

489. Quesada J, Senar JC (2007) The role of melanin- and carotenoid-based plumage coloration in nest defence in the Great Tit. Ethology 113: 640-647.

490. Quillfeldt P (2002) Begging in the absence of sibling competition in Wilson's storm-petrels, Oceanites oceanicus. Animal Behaviour 64: 579-587.

491. Quillfeldt P, Masello JF, Hamer KC (2004) Sex differences in provisioning rules and honest signalling of need in Manx shearwaters, Puffinus puffinus. Animal Behaviour 68: 613-620.

492. Quillfeldt P, Masello JF, Strange IJ, Buchanan KL (2006) Begging and provisioning of thin-billed prions, Pachyptila belcheri, are related to testosterone and corticosterone. Animal Behaviour 71: 1359-1369.

493. Quillfeldt P, Trager I, Griffiths K, Buchanan KL, Masello JF (2007) Is sex-specific mass gain in Cory's shearwaters Calonectris diomedea related to begging and steroid hormone expression? Behavioral Ecology and Sociobiology 61: 793-800.

494. Radford AN (2008) Age-related changes in nestling diet of the cooperatively breeding green woodhoopoe. Ethology 114: 907-915.

495. Radford AN (2009) Selective Prey Delivery to Incubating Females by Dominant Males, but not Helpers, in the Cooperatively Breeding Green Woodhoopoe. Ethology 115: 1082-1090.

496. Raihani NJ, Ridley AR (2007) Adult vocalizations during provisioning: offspring response and postfledging benefits in wild pied babblers. Animal Behaviour 74: 1303-1309.

497. Rapaport LG, Ruiz-Miranda CR (2006) Ontogeny of provisioning in two populations of wild golden lion tamarins (Leontopithecus rosalia). Behavioral Ecology and Sociobiology 60: 724-735.

498. Rastogi AD, Zanette L, Clinchy M (2006) Food availability affects diurnal nest predation and adult antipredator behaviour in song sparrows, Melospiza melodia. Animal Behaviour 72: 933-940.

499. Ratikainen, II, Kokko H (2010) Differential allocation and compensation: who deserves the silver spoon? Behavioral Ecology 21: 195-200.

500. Rauter CM, McGuire MJ, Gwartney MM, Space JE (2010) Effect of Population Density and Female Body Size on Number and Size of Offspring in a Species with Size-Dependent Contests over Resources. Ethology 116: 120-128.

501. Rauter CM, Moore AJ (2004) Time constraints and trade-offs among parental care behaviours: effects of brood size, sex and loss of mate. Animal Behaviour 68: 695-702.

502. Redpath SM, Leckie FM, Arroyo B, Amar A, Thirgood SJ (2006) Compensating for the costs of polygyny in hen harriers Circus cyaneus. Behavioral Ecology and Sociobiology 60: 386-391.

503. Reguera P, Gomendio M (2002) Flexible oviposition behavior in the golden egg bug (Phyllomorpha laciniata) and its implications for offspring survival. Behavioral Ecology 13: 70-74.

504. Rehling A, Trillmich F (2007) Weaning in the guinea pig (Cavia aperea f. porcellus): Who decides and by what measure? Behavioral Ecology and Sociobiology 62: 149-157.

505. Rehling A, Trillmich F (2008) Changing supply and demand by cross-fostering: effects on the behaviour of pups and mothers in guinea pigs, Cavia aperea f. porcellus, and cavies, Cavia aperea. Animal Behaviour 75: 1455-1463.

506. Reid JM, Monaghan P, Ruxton GD (2002) Males matter: The occurrence and consequences of male incubation in starlings (Sturnus vulgaris). Behavioral Ecology and Sociobiology 51: 255-261.

507. Remes V (2005) Nest concealment and parental behaviour interact in affecting nest survival in the blackcap (Sylvia atricapilla): an experimental evaluation of the parental compensation hypothesis. Behavioral Ecology and Sociobiology 58: 326-332.

508. Rensel MA, Wilcoxen TE, Schoech SJ (2010) The influence of nest attendance and provisioning on nestling stress physiology in the Florida scrub-jay. Hormones and Behavior 57: 162-168.

509. Requena GS, Buzatto BA, Munguia-Steyer R, Machado G (2009) Efficiency of uniparental male and female care against egg predators in two closely related syntopic harvestmen. Animal Behaviour 78: 1169-1176.

510. Ridley AR, Raihani NJ (2007) Variable postfledging care in a cooperative bird: causes and consequences. Behavioral Ecology 18: 994-1000.

511. Ridley AR, Raihani NJ (2008) Task partitioning increases reproductive output in a cooperative bird. Behavioral Ecology 19: 1136-1142.

512. Rios-Cardenas O, Webster AS (2005) Paternity and paternal effort in the pumpkinseed sunfish. Behavioral Ecology 16: 914-921.

513. Riou S, Hamer KC (2008) Predation risk and reproductive effort: impacts of moonlight on food provisioning and chick growth in Manx shearwaters. Animal Behaviour 76: 1743-1748.

514. Rivers JW (2007) Nest mate size, but not short-term need, influences begging behavior of a generalist brood parasite. Behavioral Ecology 18: 222-230.

515. Rivers JW (2009) Parent-absent begging in the Brown-headed Cowbird (Molothrus ater): the role of short-term need and nestmate size. Behavioral Ecology and Sociobiology 63: 707-717.

516. Rivers JW, Briskie JV, Rothstein SI (2010) Have brood parasitic cowbird nestlings caused the evolution of more intense begging by host nestlings? Animal Behaviour 80: E1-E5.

517. Rivers JW, Loughin TM, Rothstein SI (2010) Brown-headed cowbird nestlings influence nestmate begging, but not parental feeding, in hosts of three distinct sizes. Animal Behaviour 79: 107-116.

518. Roberts RL, Jenkins KT, Lawler T, Wegner FH, Newman JD (2001) Bromocriptine administration lowers serum prolactin and disrupts parental responsiveness in common marmosets (Callithrix j. jacchus). Hormones and Behavior 39: 106-112.

519. Rodgers EW, Earley RL, Grober MS (2006) Elevated 11-ketotestosterone during paternal behavior in the Bluebanded goby (Lythrypnus dalli). Hormones and Behavior 49: 610-614.

520. Rodriguez-Girones MA, Enquist M, Lachmann M (2001) Role of begging and sibling competition in foraging strategies of nestlings. Animal Behaviour 61: 733-745.

521. Rodriguez-Girones MA, Zuniga JM, Redondo T (2001) Effects of begging on growth rates of nestling chicks. Behavioral Ecology 12: 269-274.

522. Rodriguez-Girones MA, Zuniga JM, Redondo T (2002) Feeding experience and relative size modify the begging strategies of nestlings. Behavioral Ecology 13: 782-785.

523. Roper A, Zann R (2006) The onset of song learning and song tutor selection in fledgling zebra finches. Ethology 112: 458-470.

524. Ros AFH, Bruintjes R, Santos RS, Canario AVM, Oliveira RF (2004) The role of androgens in the trade-off between territorial and parental behavior in the Azorean rock-pool blenny, Parablennius parvicornis. Hormones and Behavior 46: 491-497.

525. Ros AFH, Canario AVM, Couto E, Zeilstra I, Oliveira RF (2003) Endocrine correlates of intra-specific variation in the mating system of the St. Peter's fish (Sarotherodon galilaeus). Hormones and Behavior 44: 365-373.

526. Ros AFH, Fagundes T, Oliveira RF (2009) Adjustment of brood size and androgen levels in a teleost species with exclusive male parental care. Animal Behaviour 78: 25-33.

527. Rosvall KA (2010) Do males offset the cost of female aggression? An experimental test in a biparental songbird. Behavioral Ecology 21: 161-168.

528. Roulin A (2001) Food supply differentially affects sibling negotiation and competition in the barn owl (Tyto alba). Behavioral Ecology and Sociobiology 49: 514-519.

529. Roulin A (2001) On the cost of begging vocalization: implications of vigilance. Behavioral Ecology 12: 506-511.

530. Roulin A (2002) Why do lactating females nurse alien offspring? A review of hypotheses and empirical evidence. Animal Behaviour 63: 201-208.

531. Roulin A, Bersier LF (2007) Nestling barn owls beg more intensely in the presence of their mother than in the presence of their father. Animal Behaviour 74: 1099-1106.

532. Roulin A, Dreiss AN, Kolliker M (2010) Evolutionary Perspective on the Interplay Between Family Life, and Parent and Offspring Personality. Ethology 116: 787-796.

533. Royle NJ, Hartley IR, Parker GA (2006) Consequences of biparental care for begging and growth in zebra finches, Taeniopygia guttata. Animal Behaviour 72: 123-130.

534. Royle NJ, Schuett W, Dall SRX (2010) Behavioral consistency and the resolution of sexual conflict over parental investment. Behavioral Ecology 21: 1125-1130.

535. Ruscio MG, Adkins-Regan E (2004) Immediate early gene expression associated with induction of brooding behavior in Japanese quail. Hormones and Behavior 46: 19-29.

536. Russell EM, Yom-Tov Y, Geffen E (2004) Extended parental care and delayed dispersal: northern, tropical, and southern passerines compared. Behavioral Ecology 15: 831-838.

537. Ruusila V, Poysa H, Runko P (2001) Costs and benefits of female-biased natal philopatry in the common goldeneye. Behavioral Ecology 12: 686-690.

538. Ruuskanen S, Doligez B, Tschirren B, Pitala N, Gustafsson L, et al. (2009) Yolk androgens do not appear to mediate sexual conflict over parental investment in the collared flycatcher Ficedula albicollis. Hormones and Behavior 55: 514-519.

539. Rymer T, Schradin C, Pillay N (2008) Social transmission of information about novel food in two populations of the African striped mouse, Rhabdomys pumilio. Animal Behaviour 76: 1297-1304.

540. Rytkonen S (2002) Nest defence in great tits Parus major: support for parental investment theory. Behavioral Ecology and Sociobiology 52: 379-384.

541. Sacchi R, Saino N, Galeotti P (2002) Features of begging calls reveal general condition and need of food of barn swallow (Hirundo rustica) nestlings. Behavioral Ecology 13: 268-273.

542. Safran RJ, Pilz KM, McGraw KJ, Correa SM, Schwabl H (2008) Are yolk androgens and carotenoids in barn swallow eggs related to parental quality? Behavioral Ecology and Sociobiology 62: 427-438.

543. Saino N, Ambrosini R, Martinelli R, Ninni P, Moller AP (2003) Gape coloration reliably reflects immunocompetence of barn swallow (Hirundo rustica) nestlings. Behavioral Ecology 14: 16-22.

544. Saino N, De Ayala RM, Boncoraglio G, Martinelli R (2008) Sex difference in mouth coloration and begging calls of barn swallow nestlings. Animal Behaviour 75: 1375-1382.

545. Saino N, Galeotti P, Sacchi R, Boncoraglio G, Martinelli R, et al. (2003) Sex differences in begging vocalizations of nestling barn swallows, Hirundo rustica. Animal Behaviour 66: 1003-1010.

546. Saino N, Incagli M, Martinelli R, Moller AP (2002) Immune response of male barn swallows in relation to parental effort, corticosterone plasma levels, and sexual ornamentation. Behavioral Ecology 13: 169-174.

547. Saino N, Romano M, Rubolini D, Caprioli M, Ambrosini R, et al. (2010) Food supplementation affects egg albumen content and body size asymmetry among yellow-legged gull siblings. Behavioral Ecology and Sociobiology 64.

548. Salomon M, Lubin Y (2007) Cooperative breeding increases reproductive success in the social spider Stegodyphus dumicola (Araneae, Eresidae). Behavioral Ecology and Sociobiology 61: 1743-1750.

549. Samelius G, Alisauskas RT (2006) Sex-biased costs in nest defence behaviours by lesser snow geese (Chen caerulescens): consequences of parental roles? Behavioral Ecology and Sociobiology 59: 805-810.

550. Samuel PA, Bales KL (2005) Effects of urocortin II on parental care, anxiety, and neuroendocrine hormones in prairie voles (microtus ochrogaster). Hormones and Behavior 48: 207.

551. Sanz JJ (2001) Experimentally reduced male attractiveness increases parental care in the pied flycatcher Ficedula hypoleuca. Behavioral Ecology 12: 171-176.

552. Sasvari L, Peczely P, Hegyi Z (2004) The influence of parental age and weather on testosterone concentration and offspring survival in broods of tawny owl Strix aluco. Behavioral Ecology and Sociobiology 56: 306-313.

553. Sauter A, Bowman R, Schoech SJ, Pasinelli G (2006) Does optimal foraging theory explain why suburban Florida scrub-jays (Aphelocoma coerulescens) feed their young human-provided food? Behavioral Ecology and Sociobiology 60: 465-474.

554. Scaggiante M, Grober MS, Lorenzi V, Rasotto MB (2004) Changes along the male reproductive axis in response to social context in a gonochoristic gobiid, Zosterisessor ophiocephalus (Teleostei, Gobiidae), with alternative mating tactics. Hormones and Behavior 46: 607-617.

555. Schielzeth H, Bolund E (2010) Patterns of conspecific brood parasitism in zebra finches. Animal Behaviour 79: 1329-1337.

556. Schradin C (2007) Comments to K.E. Wynne-Edwards and M.E. Timonin 2007. Paternal care in rodents: Weakening support of hormonal regulation of the transition to behavioral fatherhood in rodent animal models of biparental care, Horm & Behav 52 : 114-121. Hormones and Behavior 52: 557-559.

557. Schradin C, Anzenberger G (2004) Development of prolactin levels in marmoset males: From adult son to first-time father. Hormones and Behavior 46: 670-677.

558. Schradin C, Pillay N (2004) Prolactin levels in paternal striped mouse (Rhabdomys pumilio) fathers. Physiology & Behavior 81: 43-50.

559. Schradin C, Pillay N (2005) The influence of the father on offspring development in the striped mouse. Behavioral Ecology 16: 450-455.

560. Schuetz JG (2005) Common waxbills use carnivore scat to reduce the risk of nest predation. Behavioral Ecology 16: 133-137.

561. Schuetz JG (2005) Reduced growth but not survival of chicks with altered gape patterns: implications for the evolution of nestling similarity in a parasitic finch. Animal Behaviour 70: 839-848.

562. Schum JE, Wynne-Edwards KE (2005) Estradiol and progesterone in paternal and non-paternal hamsters (Phodopus) becoming fathers: conflict with hypothesized roles. Hormones and Behavior 47: 410-418.

563. Schwagmeyer PL, Bartlett TL, Schwabl HG (2008) Dynamics of house sparrow biparental care: What contexts trigger partial compensation? Ethology 114: 459-468.

564. Schwagmeyer PL, Mock DW (2003) How consistently are good parents good parents? Repeatability of parental care in the house sparrow, Passer domesticus. Ethology 109: 303-313.

565. Schwagmeyer PL, Mock DW, Parker GA (2002) Biparental care in house sparrows: negotiation or sealed bid? Behavioral Ecology 13: 713-721.

566. Schwagmeyer PL, Schwabl HG, Mock DW (2005) Dynamics of biparental care in house sparrows: hormonal manipulations of paternal contributions. Animal Behaviour 69: 481-488.

567. Scott MP (2006) Resource defense and juvenile honnone: The "challenge hypothesis" extended to insects. Hormones and Behavior 49: 276-281.

568. Scott MP, Panaitof SC (2004) Social stimuli affect juvenile hormone during breeding in biparental burying beetles (Silphidae : Nicrophorus). Hormones and Behavior 45: 159-167.

569. Searby A, Jouventin P, Aubin T (2004) Acoustic recognition in macaroni penguins: an original signature system. Animal Behaviour 67: 615-625.

570. Seidelmann K (2006) Open-cell parasitism shapes maternal investment patterns in the Red Mason bee Osmia rufa. Behavioral Ecology 17: 839-848.

571. Sharpe F, Rosell F (2003) Time budgets and sex differences in the Eurasian beaver. Animal Behaviour 66: 1059-1067.

572. Shealer DA, Spendelow JA, Hatfield JS, Nisbet ICT (2005) The adaptive significance of stealing in a marine bird and its relationship to parental quality. Behavioral Ecology 16: 371-376.

573. Shreeves G, Field J (2008) Parental care and sexual size dimorphism in wasps and bees. Behavioral Ecology and Sociobiology 62: 843-852.

574. Siefferman L, Hill GE (2003) Structural and melanin coloration indicate parental effort and reproductive success in male eastern bluebirds. Behavioral Ecology 14: 855-861.

575. Siefferman L, Hill GE (2007) The effect of rearing environment on blue structural coloration of eastern bluebirds (Sialia sialis). Behavioral Ecology and Sociobiology 61: 1839-1846.

576. Silva MC, Boersma PD, Mackay S, Strange I (2007) Egg size and parental quality in thin-billed prions, Pachyptila belcheri: effects on offspring fitness. Animal Behaviour 74: 1403-1412.

577. Silva N, Aviles JM, Danchin E, Parejo D (2008) Informative content of multiple plumage-coloured traits in female and male European Rollers. Behavioral Ecology and Sociobiology 62: 1969-1979.

578. Simoncelli LA, Delevan CJ, Al-Naimi OAS, Bamshad M (2010) Female tactile cues maximize paternal behavior in prairie voles. Behavioral Ecology and Sociobiology 64: 865-873.

579. Sinn DL, While GM, Wapstra E (2008) Maternal care in a social lizard: links between female aggression and offspring fitness. Animal Behaviour 76: 1249-1257.

580. Slagsvold T, Wiebe KL (2007) Hatching asynchrony and early nestling mortality: the feeding constraint hypothesis. Animal Behaviour 73: 691-700.

581. Smiseth PT, Andrews C, Brown E, Prentice PM (2010) Chemical stimuli from parents trigger larval begging in burying beetles. Behavioral Ecology 21: 526-531.

582. Smiseth PT, Bu RJ, Eikenaes AK, Amundsen T (2003) Food limitation in asynchronous bluethroat broods: effects on food distribution, nestling begging, and parental provisioning rules. Behavioral Ecology 14: 793-801.

583. Smiseth PT, Dawson C, Varley E, Moore AJ (2005) How do caring parents respond to mate loss? Differential response by males and females. Animal Behaviour 69: 551-559.

584. Smiseth PT, Lorentsen SH (2001) Begging and parent-offspring conflict in grey seals. Animal Behaviour 62: 273-279.

585. Smiseth PT, Moore AJ (2002) Does resource availability affect offspring begging and parental provisioning in a partially begging species? Animal Behaviour 63: 577-585.

586. Smiseth PT, Moore AJ (2004) Behavioral dynamics between caring males and females in a beetle with facultative biparental care. Behavioral Ecology 15: 621-628.

587. Smiseth PT, Moore AJ (2004) Signalling of hunger when offspring forage by both begging and self-feeding. Animal Behaviour 67: 1083-1088.

588. Smiseth PT, Moore AJ (2007) Signalling of hunger by senior and junior larvae in asynchronous broods of a burying beetle. Animal Behaviour 74: 699-705.

589. Smiseth PT, Moore AJ (2008) Parental distribution of resources in relation to larval hunger and size rank in the burying beetle Nicrophorus vespilloides. Ethology 114: 789-796.

590. Smiseth PT, Morgan K (2009) Asynchronous hatching in burying beetles: a test of the peak load reduction hypothesis. Animal Behaviour 77: 519-524.

591. Smiseth PT, Ornborg J, Andersson S, Amundsen T (2001) Is male plumage reflectance correlated with paternal care in bluethroats? Behavioral Ecology 12: 164-170.

592. Smiseth PT, Parker HJ (2008) Is there a cost to larval begging in the burying beetle Nicrophorus vespilloides? Behavioral Ecology 19: 1111-1115.

593. Smith TE, Leonard ML, Smith BD (2005) Provisioning rules and chick competition in asynchronously hatching common terns (Sterna hirundo). Behavioral Ecology and Sociobiology 58: 456-465.

594. Snekser JL, Itzkowitz M (2009) Sex Differences in Retrieval Behavior by the Biparental Convict Cichlid. Ethology 115: 457-464.

595. Sockman KW, Schwabl H, Sharp PJ (2004) Removing the confound of time in investigating the regulation of serial behaviours: testosterone, prolactin and the transition from sexual to parental activity in male American kestrels. Animal Behaviour 67: 1151-1161.

596. Soler JJ, Aviles JM, Cuervo JJ, Perez-Contreras T (2007) Is the relation between colour and immune response mediated by nutritional condition in spotless starling nestlings? Animal Behaviour 74: 1139-1145.

597. Soler JJ, de Neve L, Martinez JG, Soler M (2001) Nest size affects clutch size and the start of incubation in magpies: an experimental study. Behavioral Ecology 12: 301-307.

598. Soler M (2008) Do hosts of interspecific brood parasites feed parasitic chicks with lower-quality prey? Animal Behaviour 76: 1761-1763.

599. Soltis J, Wegner FH, Newman JD (2005) Urinary prolactin is correlated with mothering and allo-mothering in squirrel monkeys. Physiology & Behavior 84: 295-301.

600. Soma M, Saito DS, Hasegawa T, Okanoya K (2007) Sex-specific maternal effect on egg mass, laying order, and sibling competition in the Bengalese finch (Lonchura striata var. domestica). Behavioral Ecology and Sociobiology 61: 1695-1705.

601. Spee M, Beaulieu M, Dervaux A, Chastel O, Le Maho Y, et al. (2010) Should I stay or should I go? Hormonal control of nest abandonment in a long-lived bird, the Adelie penguin. Hormones and Behavior 58: 762-768.

602. Spencer KA, Heidinger BJ, D'Alba LB, Evans NP, Monaghan P (2010) Then versus now: effect of developmental and current environmental conditions on incubation effort in birds. Behavioral Ecology 21: 999-1004.

603. Spoon TR, Millam JR, Owings DH (2006) The importance of mate behavioural compatibility in parenting and reproductive success by cockatiels, Nymphicus hollandicus. Animal Behaviour 71: 315-326.

604. St Mary CM, Noureddine CG, Lindstrom K (2001) Environmental effects on male reproductive success and parental care in the Florida flagfish Jordanella floridae. Ethology 107: 1035-1052.

605. Staerkle M, Kolliker M (2008) Maternal food regurgitation to nymphs in earwigs (Forficula auricularia). Ethology 114: 844-850.

606. Stahlschmidt ZR, DeNardo DF (2009) Effect of nest temperature on egg-brooding dynamics in Children's pythons. Physiology & Behavior 98: 302-306.

607. Stahlschmidt ZR, Hoffman TCM, DeNardo DF (2008) Postural Shifts During Egg-Brooding and Their Impact on Egg Water Balance in Children's Pythons (Antaresia childreni). Ethology 114: 1113-1121.

608. Stankowich T, Sherman PW (2002) Pup shoving by adult naked mole-rats. Ethology 108: 975-992.

609. Steen R, Low LM, Sonerud GA, Selas V, Slagsvold T (2010) The feeding constraint hypothesis: prey preparation as a function of nestling age and prey mass in the Eurasian kestrel. Animal Behaviour 80: 147-153.

610. Steer J, Burns KC (2008) Seasonal variation in male-female competition, cooperation and selfish hoarding in a monogamous songbird. Behavioral Ecology and Sociobiology 62: 1175-1183.

611. Steiger S, Muller JK (2010) From class-specific to individual discrimination: acceptance threshold changes with risk in the partner recognition system of the burying beetle Nicrophorus vespilloides. Animal Behaviour 80: 607-613.

612. Steinegger M, Taborsky B (2007) Asymmetric sexual conflict over parental care in a biparental cichlid. Behavioral Ecology and Sociobiology 61: 933-941.

613. Steinhart GB, Sandrene ME, Weaver S, Stein RA, Marschall EA (2005) Increased parental care cost for nest-guarding fish in a lake with hyperabundant nest predators. Behavioral Ecology 16: 427-434.

614. Stiver KA, Alonzo SH (2009) Parental and Mating Effort: Is There Necessarily a Trade-Off? Ethology 115: 1101-1126.

615. Strohm E, Marliani A (2002) The cost of parental care: prey hunting in a digger wasp. Behavioral Ecology 13: 52-58.

616. Stynoski JL (2009) Discrimination of offspring by indirect recognition in an egg-feeding dendrobatid frog, Oophaga pumilio. Animal Behaviour 78: 1351-1356.

617. Suk HY, Choe JC (2002) The presence of eggs in the nest and female choice in common freshwater gobies (Rhinogobius brunneus). Behavioral Ecology and Sociobiology 52: 211-215.

618. Suryanarayanan S, Jeanne RL (2008) Antennal Drumming, Trophallaxis, and Colony Development in the Social Wasp Polistes fuscatus (Hymenoptera: Vespidae). Ethology 114: 1201-1209.

619. Suter SM, Bielanska J, Rothlin-Spillmann S, Strambini L, Meyer DR (2009) The cost of infidelity to female reed buntings. Behavioral Ecology 20: 601-608.

620. Suzuki S, Nagano M (2009) To Compensate or Not? Caring Parents Respond Differentially to Mate Removal and Mate Handicapping in the Burying Beetle, Nicrophorus quadripunctatus. Ethology 115: 1-6.

621. Svensson O, Lissaker M, Mobley KB (2010) Offspring recognition and the influence of clutch size on nest fostering among male sand gobies, Pomatoschistus minutus. Behavioral Ecology and Sociobiology 64: 1325-1331.

622. Swaisgood RR, Rowe MP, Owings DH (2003) Antipredator responses of California ground squirrels to rattlesnakes and rattling sounds: the roles of sex, reproductive parity, and offspring age in assessment and decision-making rules. Behavioral Ecology and Sociobiology 55: 22-31.

623. Szabo A, Duffus D (2008) Mother-offspring association in the humpback whale, Megaptera novaeangliae: following behaviour in an aquatic mammal. Animal Behaviour 75: 1085-1092.

624. Szentirmai I, Komdeur J, Szekely T (2005) What makes a nest-building male successful? Male behavior and female care in penduline tits. Behavioral Ecology 16: 994-1000.

625. Taborsky B, Foerster K (2004) Female mouthbrooders adjust incubation duration to perceived risk of predation. Animal Behaviour 68: 1275-1281.

626. Taborsky B, Skubic E, Bruintjes R (2007) Mothers adjust egg size to helper number in a cooperatively breeding cichlid. Behavioral Ecology 18: 652-657.

627. Takahashi D, Kohda M (2004) Courtship in fast water currents by a male stream goby (Rhinogobius brunneus) communicates the parental quality honestly. Behavioral Ecology and Sociobiology 55: 431-438.

628. Tanner M, Kolliker M, Richner H (2007) Parental influence on sibling rivalry in great tit, Parus major, nests. Animal Behaviour 74: 977-983.

629. Tanner M, Kolliker M, Richner H (2008) Differential food allocation by male and female great tit, Parus major, parents: are parents or offspring in control? Animal Behaviour 75: 1563-1569.

630. Tanner M, Richner H (2008) Ultraviolet reflectance of plumage for parent-offspring communication in the great tit (Parus major). Behavioral Ecology 19: 369-373.

631. Tardif SD, Layne DG, Smucny DA (2002) Can marmoset mothers count to three? Effect of litter size on mother-infant interactions. Ethology 108: 825-836.

632. Tarwater CE, Brawn JD (2008) Patterns of brood division and an absence of behavioral plasticity in a neotropical passerine. Behavioral Ecology and Sociobiology 62: 1441-1452.

633. Tarwater CE, Brawn JD (2010) Family living in a Neotropical bird: variation in timing of dispersal and higher survival for delayed dispersers. Animal Behaviour 80: 535-542.

634. Tarwater CE, Kelley JP, Brawn JD (2009) Parental response to elevated begging in a high predation, tropical environment. Animal Behaviour 78: 1239-1245.

635. Tay WT, Miettinen M, Kaitala A (2003) Do male golden egg bugs carry eggs they have fertilized? A microsatellite analysis. Behavioral Ecology 14: 481-485.

636. Teichroeb JA, Sicotte P (2008) Social correlates of fecal testosterone in male ursine colobus monkeys (Colobus vellerosus): The effect of male reproductive competition in aseasonal breeders. Hormones and Behavior 54: 417-423.

637. Thomas LK, Manica A (2003) Filial cannibalism in an assassin bug. Animal Behaviour 66: 205-210.

638. Thomas LK, Manica A (2005) Intrasexual competition and mate choice in assassin bugs with uniparental male and female care. Animal Behaviour 69: 275-281.

639. Thunken T, Meuthen D, Bakker TCM, Kullmann H (2010) Parental investment in relation to offspring quality in the biparental cichlid fish Pelvicachromis taeniatus. Animal Behaviour 80: 69-74.

640. Tieleman BI, Dijkstra TH, Klasing KC, Visser GH, Williams JB (2008) Effects of experimentally increased costs of activity during reproduction on parental investment and self-maintenance in tropical house wrens. Behavioral Ecology 19: 949-959.

641. Timonin ME, Wynne-Edwards KE (2006) Neither reduced photoperiod, nor female-related social cues, nor increased maternal thermal stress result in a paternally responsive Phodopus sungorus male. Physiology & Behavior 88: 309-316.

642. Timonin ME, Wynne-Edwards KE (2008) Aromatase inhibition during adolescence reduces adult sexual and paternal behavior in the biparental dwarf hamster Phodopus campbelli. Hormones and Behavior 54: 748-757.

643. Tinbergen JM, Sanz JJ (2004) Strong evidence for selection for larger brood size in a great tit population. Behavioral Ecology 15: 525-533.

644. Tinkler E, Montgomery WI, Elwood RW (2007) Shared or unshared parental care in overwintering brent geese (Branta bernicla hrota). Ethology 113: 368-376.

645. Tobler M, Smith HG (2010) Mother-offspring conflicts, hormone signaling, and asymmetric ownership of information. Behavioral Ecology 21: 893-897.

646. Torok J, Hargitai R, Hegyi G, Matus Z, Michl G, et al. (2007) Carotenoids in the egg yolks of collared flycatchers (Ficedula albicollis) in relation to parental quality, environmental factors and laying order. Behavioral Ecology and Sociobiology 61: 541-550.

647. Trainor BC, Finy MS, Nelson RJ (2008) Paternal aggression in a biparental mouse: Parallels with maternal aggression. Hormones and Behavior 53: 200-207.

648. Trainor BC, Marler CA (2001) Testosterone, paternal behavior, and aggression in the monogamous California mouse (Peromyscus californicus). Hormones and Behavior 40: 32-42.

649. Treves A, Drescher A, Snowdon CT (2003) Maternal watchfulness in black howler monkeys (Alouatta pigra). Ethology 109: 135-146.

650. Trillmich F, Wolf JBW (2008) Parent-offspring and sibling conflict in Galapagos fur seals and sea lions. Behavioral Ecology and Sociobiology 62: 363-375.

651. Trnka A, Prokop P (2010) Does Social Mating System Influence Nest Defence Behaviour in Great Reed Warbler (Acrocephalus arundinaceus) Males? Ethology 116: 1075-1083.

652. Trumbo ST (2007) Defending young biparentally: female risk-taking with and without a male in the burying beetle, Nicrophorus pustulatus. Behavioral Ecology and Sociobiology 61: 1717-1723.

653. Trumbo ST (2009) Age-related reproductive performance in the parental burying beetle, Nicrophorus orbicollis. Behavioral Ecology 20: 951-956.

654. Trumbo ST, Valletta RC (2007) The costs of confronting infanticidal intruders in a burying beetle. Ethology 113: 386-393.

655. Tschirren B, Richner H (2008) Differential effects of yolk hormones on maternal and paternal contribution to parental care. Animal Behaviour 75: 1989-1994.

656. Vaclav R, Hoi H (2002) Different reproductive tactics in house sparrows signalled by badge size: Is there a benefit to being average? Ethology 108: 569-582.

657. Vaclav R, Hoi H, Blomqvist D (2003) Food supplementation affects extrapair paternity in house sparrows (Passer domesticus). Behavioral Ecology 14: 730-735.

658. Valencia J, De la Cruz C, Carranza J, Mateos C (2006) Parents increase their parental effort when aided by helpers in a cooperatively breeding bird. Animal Behaviour 71: 1021-1028.

659. van Dijk RE, Meszaros LA, van der Velde M, Szekely T, Akos P, et al. (2010) Nest desertion is not predicted by cuckoldry in the Eurasian penduline tit. Behavioral Ecology and Sociobiology 64: 1425-1435.

660. Van Duyse E, Pinxten R, Eens M (2002) Effects of testosterone on song, aggression, and nestling feeding behavior in male great tits, Parus major. Hormones and Behavior 41: 178-186.

661. Van Dyck H, Regniers S (2010) Egg spreading in the ant-parasitic butterfly, Maculinea alcon: from individual behaviour to egg distribution pattern. Animal Behaviour 80: 621-627.

662. Van Opzeeland IC, Van Parijs SM (2004) Individuality in harp seal, Phoca groenlandica, pup vocalizations. Animal Behaviour 68: 1115-1123.

663. Van Roo BL (2004) Exogenous testosterone inhibits several forms of male parental behavior and stimulates song in a monogamous songbird: The blue-headed vireo (Vireo solitarius). Hormones and Behavior 46: 678-683.

664. Van Roo BL, Ketterson ED, Sharp PJ (2003) Testosterone and prolactin in two songbirds that differ in paternal care: the blue-headed vireo and the red-eyed vireo. Hormones and Behavior 44: 435-441.

665. Vasconcelos RO, Simoes JM, Almada VC, Fonseca PJ, Amorim MCP (2010) Vocal Behavior During Territorial Intrusions in the Lusitanian Toadfish: Boatwhistles Also Function as Territorial 'Keep-Out' Signals. Ethology 116: 155-165.

666. Vega LB, Holloway GJ, Millett JE, Richardson DS (2007) Extreme gender-based post-fledging brood division in the toc-toc. Behavioral Ecology 18: 730-735.

667. Velando A (2002) Experimental manipulation of maternal effort produces differential effects in sons and daughters: implications for adaptive sex ratios in the blue-footed booby. Behavioral Ecology 13: 443-449.

668. Velez MJ, Jennions MD, Telford SR (2002) The effect of an experimental brood reduction on male desertion in the Panamanian blue acara Cichlid Aequidens coeruleopunctatus. Ethology 108: 331-340.

669. Verboven N, Tinbergen JM (2002) Nest desertion: a trade-off between current and future reproduction. Animal Behaviour 63: 951-958.

670. Verboven N, Verreault J, Letcher RJ, Gabrielsen GW, Evans NP (2009) Nest temperature and parental behaviour of Arctic-breeding glaucous gulls exposed to persistent organic pollutants. Animal Behaviour 77: 411-418.

671. Vergara P, Fargallo JA (2008) Sex, melanic coloration, and sibling competition during the postfledging dependence period. Behavioral Ecology 19: 847-853.

672. Vergara P, Fargallo JA, Martinez-Padilla J (2010) Reaching independence: food supply, parent quality, and offspring phenotypic characters in kestrels. Behavioral Ecology 21: 507-512.

673. Villasenor E, Drummond H (2007) Honest begging in the blue-footed booby: signaling food deprivation and body condition. Behavioral Ecology and Sociobiology 61: 1133-1142.

674. Voltura KM, Schwagmeyer PL, Mock DW (2002) Parental feeding rates in the house sparrow, Passer domesticus: Are larger-badged males better fathers? Ethology 108: 1011-1022.

675. Walling CA, Stamper CE, Salisbury CL, Moore AJ (2009) Experience does not alter alternative mating tactics in the burying beetle Nicrophorus vespilloides. Behavioral Ecology 20: 153-159.

676. Ward RJS, Cotter SC, Kilner RM (2009) Current brood size and residual reproductive value predict offspring desertion in the burying beetle Nicrophorus vespilloides. Behavioral Ecology 20: 1274-1281.

677. Warner DA, Shine R (2008) Maternal nest-site choice in a lizard with temperature-dependent sex determination. Animal Behaviour 75: 861-870.

678. Washabaugh KF, Snowdon CT, Ziegler TE (2002) Variations in care for cottontop tamarin, Saguinus oedipus, infants as a function of parental experience and group size. Animal Behaviour 63: 1163-1174.

679. Wedell N, Kvarnemo C, Lessells CKM, Tregenza T (2006) Sexual conflict and life histories. Animal Behaviour 71: 999-1011.

680. Weladji RB, Holand O, Steinheim G, Lenvik D (2003) Sex-specific preweaning maternal care in reindeer (Rangifer tarandus t.). Behavioral Ecology and Sociobiology 53: 308-314.

681. Wesolowski T (2004) The origin of parental care in birds: a reassessment. Behavioral Ecology 15: 520-523.

682. Wheeler BC (2008) Selfish or altruistic? An analysis of alarm call function in wild capuchin monkeys, Cebus apella nigritus. Animal Behaviour 76: 1465-1475.

683. Wheelwright NT, Tice KA, Freeman-Gallant CR (2003) Postfledging parental care in Savannah sparrows: sex, size and survival. Animal Behaviour 65: 435-443.

684. While GM, Uller T, Wapstra E (2009) Family conflict and the evolution of sociality in reptiles. Behavioral Ecology 20: 245-250.

685. While GM, Wapstra E (2009) Effects of basking opportunity on birthing asynchrony in a viviparous lizard. Animal Behaviour 77: 1465-1470.

686. White PA (2008) Maternal response to neonatal sibling conflict in the spotted hyena, Crocuta crocuta. Behavioral Ecology and Sociobiology 62: 353-361.

687. Whittingham LA, Dunn PO, Clotfelter ED (2003) Parental allocation of food to nestling tree swallows: the influence of nestling behaviour, sex and paternity. Animal Behaviour 65: 1203-1210.

688. Wiebe KL (2004) Innate and learned components of defence by flickers against a novel nest competitor, the European starling. Ethology 110: 779-791.

689. Wiebe KL (2005) Asymmetric costs favor female desertion in the facultatively polyandrous northern flicker (Colaptes auratus): a removal experiment. Behavioral Ecology and Sociobiology 57: 429-437.

690. Wiebe KL, Kempenaers B (2009) The social and genetic mating system in flickers linked to partially reversed sex roles. Behavioral Ecology 20: 453-458.

691. Wiebe KL, Slagsvold T (2009) Mouth coloration in nestling birds: increasing detection or signalling quality? Animal Behaviour 78: 1413-1420.

692. Wiebe KL, Slagsvold T (2009) Parental Sex Differences in Food Allocation to Junior Brood Members as Mediated by Prey Size. Ethology 115: 49-58.

693. Wilcoxen TE, Boughton RK, Schoech SJ (2010) Older can be better: physiological costs of paternal investment in the Florida scrub-jay. Behavioral Ecology and Sociobiology 64: 1527-1535.

694. Wisenden BD, Snekser JL, Stumbo AD, Leese JM (2008) Parental defence of an empty nest after catastrophic brood loss. Animal Behaviour 76: 2059-2067.

695. Wisenden B, Dye T (2009) Young convict cichlids use visual information to update olfactory homing cues. Behavioral Ecology and Sociobiology 63: 443-449.

696. Woodward K, Richards MH (2005) The parental investment model and minimum mate choice criteria in humans. Behavioral Ecology 16: 57-61.

697. Woolfenden BE, Gibbs HL, Sealy SG, McMaster DG (2003) Host use and fecundity of individual female brown-headed cowbirds. Animal Behaviour 66: 95-106.

698. Woxvold IA, Mulder RA, Magrath MJL (2006) Contributions to care vary with age, sex, breeding status and group size in the cooperatively breeding apostlebird. Animal Behaviour 72: 63-73.

699. Wright HWY (2006) Paternal den attendance is the best predictor of offspring survival in the socially monogamous bat-eared fox. Animal Behaviour 71: 503-510.

700. Wright J, Hinde C, Fazey I, Both C (2002) Begging signals more than just short-term need: cryptic effects of brood size in the pied flycatcher (Ficedula hypoleuca). Behavioral Ecology and Sociobiology 52: 74-83.

701. Wright J, Karasov WH, Kazem AJN, Braga Goncalves I, McSwan E (2010) Begging and digestive responses to differences in long-term and short-term need in nestling pied flycatchers. Animal Behaviour 80: 517-525.

702. Wynne-Edwards KE (2001) Hormonal changes in mammalian fathers. Hormones and Behavior 40: 139-145.

703. Wynne-Edwards KE (2007) Reply to Schradin's letter to the editor. Hormones and Behavior 52: 560-560.

704. Wynne-Edwards KE, Timonin ME (2007) Paternal care in rodents: Weakening support for hormonal regulation of the transition to behavioral fatherhood in rodent animal models of biparental care. Hormones and Behavior 52: 114-121.

705. Young AJ, Carlson AA, Clutton-Brock T (2005) Trade-offs between extraterritorial prospecting and helping in a cooperative mammal. Animal Behaviour 70: 829-837.

706. Zbinden M, Mazzi D, Kunzler R, Largiader CR, Bakker TCM (2003) Courting virtual rivals increase ejaculate size in sticklebacks (Gasterosteus aculeatus). Behavioral Ecology and Sociobiology 54: 205-209.

707. Ziegler TE, Prudom SL, Zahed SR, Parlow AF, Wegner F (2009) Prolactin's mediative role in male parenting in parentally experienced marmosets (Callithrix jacchus). Hormones and Behavior 56: 436-443.

708. Ziegler TE, Sousa MBC (2002) Parent-daughter relationships and social controls on fertility in female common marmosets, Callithrix jacchus. Hormones and Behavior 42: 356-367.

709. Zink AG (2001) The optimal degree of parental care asymmetry among communal breeders. Animal Behaviour 61: 439-446.

710. Zink AG (2003) Intraspecific brood parasitism as a conditional reproductive tactic in the treehopper Publilia concava. Behavioral Ecology and Sociobiology 54: 406-415.

711. Zink AG (2003) Quantifying the costs and benefits of parental care in female treehoppers. Behavioral Ecology 14: 687-693.

712. Zink AG (2005) The dynamics of brood desertion among communally breeding females in the treehopper, Publilia concava. Behavioral Ecology and Sociobiology 58: 466-473.
